# Low Quality Evidence of Epidemiological Observational Studies on Leishmaniasis in Brazil

**DOI:** 10.1371/journal.pone.0106635

**Published:** 2014-09-08

**Authors:** Bruno Trentini, Mário Steindel, Mariel A. Marlow

**Affiliations:** 1 School of Medicine, Federal University of Santa Catarina, Florianópolis, Santa Catarina, Brazil; 2 Department of Microbiology, Immunology and Parasitology, Federal University of Santa Catarina, Florianópolis, Santa Catarina, Brazil; 3 Division of Infectious Diseases and Vaccinology, School of Public Health, University of California, Berkeley, California, United States of America; Royal Tropical Institute, Netherlands

## Abstract

**Background:**

Brazil has implemented systematic control methods for leishmaniasis for the past 30 years, despite an increase in cases and continued spread of the disease to new regions. A lack high quality evidence from epidemiological observational studies impedes the development of novel control methods to prevent disease transmission among the population. Here, we have evaluated the quality of observational studies on leishmaniasis conducted in Brazil to highlight this issue.

**Methods/Principal Findings:**

For this systematic review, all publications on leishmaniasis conducted in Brazil from January 1^st^, 2002 to December 31^st^, 2012 were screened via Preferred Reporting Items for Systematic Reviews and Meta-Analyses (PRISMA) checklist to select observational studies involving human subjects. The 283 included studies, representing only 14.1% of articles screened, were then further evaluated for quality of epidemiological methods and study design based on the STROBE (Strengthening the Reporting of Observational studies in Epidemiology) checklists. Over half of these studies were descriptive or case reports (53.4%, 151), followed by cross-sectional (20.8%, n = 59), case-control (8.5%, n = 24), and cohort (6.0%, n = 17). Study design was not stated in 46.6% (n = 181) and incorrectly stated in 17.5% (n = 24). Comparison groups were utilized in just 39.6% (n = 112) of the publications, and only 13.4% (n = 38) employed healthy controls. Majority of studies were performed at the city-level (62.9%, n = 178), in contrast with two (0.7%) studies performed at the national-level. Coauthorship networks showed the number of author collaborations rapidly decreased after three collaborations, with 70.9% (n = 659/929) of coauthors publishing only one article during the study period.

**Conclusions/Significance:**

A review of epidemiological research in Brazil revealed a major lack of quality and evidence. While certain indicators suggested research methods may have improved in the last two years, an emphasis on observational research which employs comparison groups and representative samples is urgently needed.

## Introduction

Leishmaniasis, a vector borne parasitic disease, was responsible for an estimated 3.3 million disability adjusted life years (DALYs) in 2010 alone, representing the highest burden among the neglected tropical diseases [1]. Despite the public health and economic burden of this disease, few targeted population-based control methods are available to governments of the most affected countries [2]. Being one of seven countries which contain 90% of leishmaniasis cases [2], Brazil's current control methods are centered around diagnosis and treatment of cases, indoor residual spraying of households with insecticide, and culling of infected canines in endemic locations [3]. An increase in the number of cases [3], continued outbreaks [4] and new endemic areas [5], [6] over the past 30 years has cast doubt on the effectiveness of this current policy. The most proactive of these methods, dog culling, remains controversial and no strong evidence in the literature has proven its effectiveness in significantly reducing cases [7], [8]. Development of novel effective population based control methods requires a solid base of transparent and high quality observational studies [9]. However, there remains a recurrent difficulty in identifying major evidence in the literature for developing innovative, successful interventions for leishmaniasis control [10] as well as a lack of scaling up among those found to be promising in pilot studies [11]. While the complexity of the disease has been highlighted as the main challenge [11], this paucity of strong evidence from well-designed observation research among the human population of endemic regions may also be a major contributing factor in the continued shortcomings of control program development.

To highlight this issue, we reviewed a decade of published epidemiological observational studies on leishmaniasis conducted in Brazil to critically evaluate their quality and level of evidence.

## Methods

For this study, we conducted a literature search utilizing the online databases PubMed, MedLine, SciELO and LiLACS from September 27^th^ to October 10^th^, 2013. All studies published from January 1^st^, 2002 to December 31^st^, 2012 identified by the key words “leishmaniasis” and “Brazil”, or their respective Portuguese translations “leishmaniose” and “Brasil” for searches in Latin American databases, were selected for screening.

We used an adapted version of the Preferred Reporting Items for Systematic Reviews and Meta-Analyses (PRISMA) checklist [12] to identify, select and critically evaluate relevant observational epidemiological research on leishmaniasis. The PRISMA checklist provided the outline for systematic inclusion and exclusion of articles. Inclusion criteria were 1) observational epidemiological studies on human leishmaniasis, 2) first author affiliated with a Brazilian institution and 3) publication during the study period. For the purpose of this review, descriptive studies and case reports were included as observational epidemiological studies as well. Exclusion criteria were 1) literature reviews, meeting abstracts or training material, 2) studies not performed among Brazilian populations, 3) studies not based principally on leishmaniasis, 4) modeling studies, 5) experimental studies (clinical trials, drug testing or vaccine trials, and molecular or laboratory studies without patients outcome) and 6) studies on vectors, pathogen and/or reservoirs without human subject measurements.

The following information was abstracted from the included studies: year of publication, open access availability, language availability, study period, study population, use of comparison group, use of healthy control group, study design reported, study design based on methods presented, and exposure(s) and outcome(s) of interest. Definitions and evaluation of the quality of epidemiological methods, evidence and presentation were based on the international guidelines described in the 2002 Lancet Epidemiology Series [13] and the STROBE (Strengthening the Reporting of Observational studies in Epidemiology) checklist [14]. Impact factors are based on the 2012 Journal Citation Reports (JCR) impact factor [15] for the journal where the article was published. Data analysis was performed in SAS 9.3 (SAS Institute. Inc., Cary, NC). Coauthorship network was created by exporting the Web of Science [16] database of references to BibExcel (software available at http://www8.umu.se/inforsk/Bibexcel/) [17] for bibliometric analysis. The resulting coauthor co-occurrence matrix was then imported to the software Pajek (software available at http://vlado.fmf.uni-lj.si/pub/networks/pajek/) [18] for mapping the network in the Kamada-Kawai layout.

## Results

**Figure 1:**
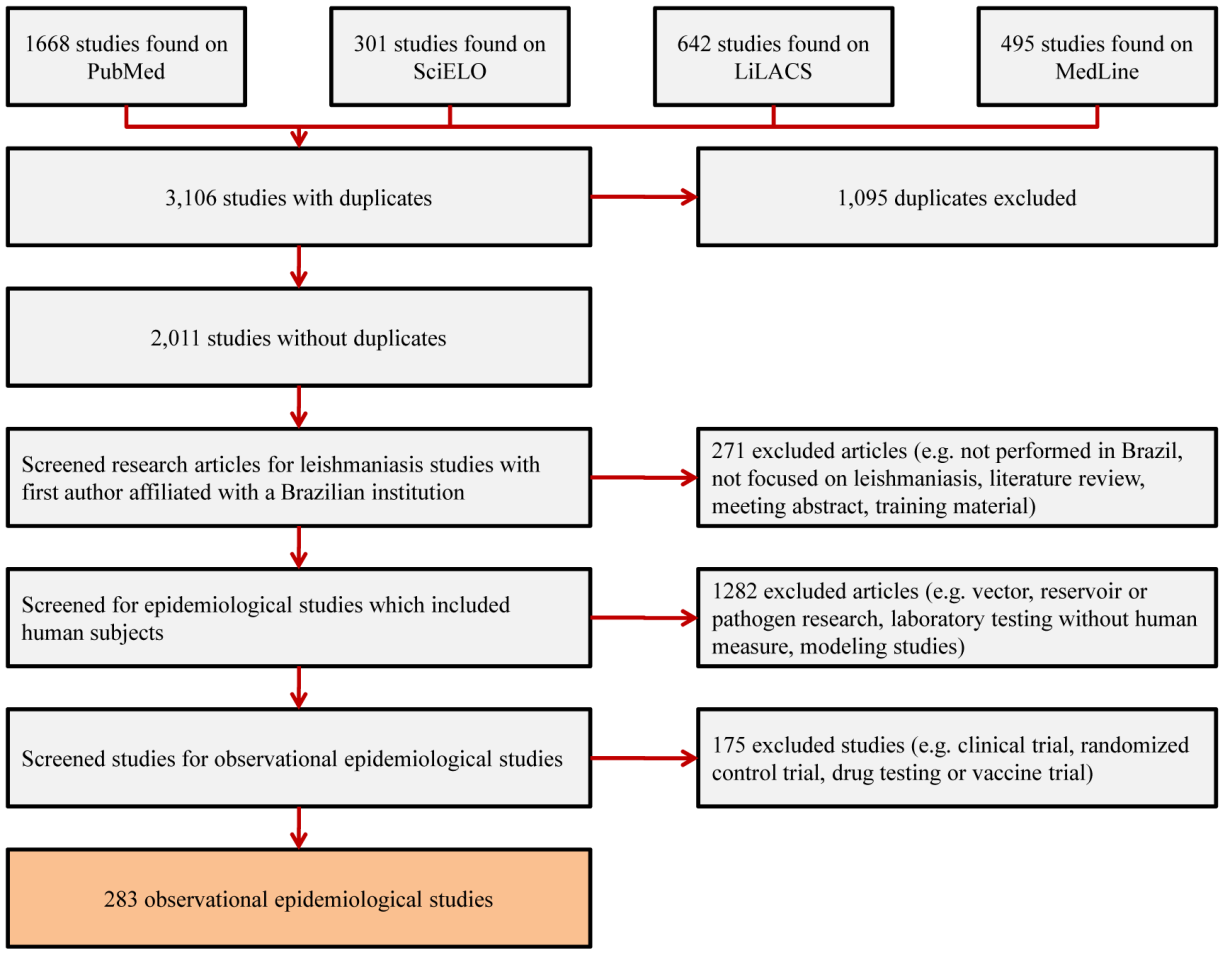
Adapted PRISMA checklist used for assessing the inclusion of articles published on epidemiological observational research on leishmaniasis involving human subjects in Brazil (2002–2012).

After deduplication, the total number of papers evaluated for inclusion through the PRISMA checklist was 2,011. Of these, 283 (14.1%) articles were found to be epidemiological observational studies performed by a Brazilian first author that incorporated human subjects and were included in the analysis (Text S1). Studies excluded were attributed to the following categories: 136 (6.8%) literature reviews or meeting abstracts, 38 (1.9%) training material, 42 (2.0%) studies not performed in a Brazilian institution or Brazil, 55 (2.7%) studies not based on leishmaniasis, 2 (0.1%) modeling studies, 12 (0.6%) clinical trials, 163 (8.1%) studies on drug testing or vaccine trials, 494 (24.5%) studies focused on molecular and laboratorial testing without patients measure, 786 (39.0%) vector, pathogen and/or reservoir studies that did not include human subjects (Figure 1).

**Figure 2:**
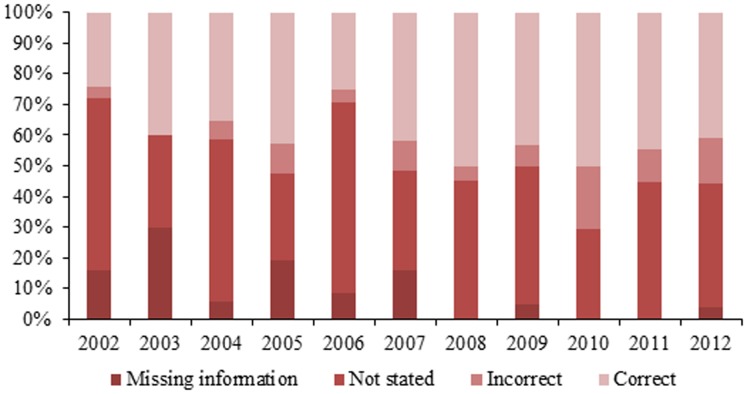
Reporting of study design in published epidemiological observational research on leishmaniasis in Brazil by year, 2002–2012.

**Figure 3:**
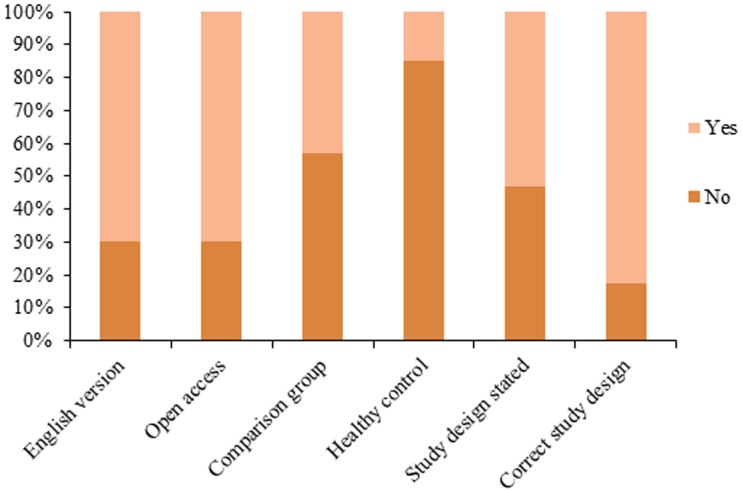
Characteristics associated with level of evidence and accessibility of published epidemiological observational research on leishmaniasis in Brazil, 2002–2012.

During the study period, an average of 28.3 studies per year were published, with the highest number of studies (n = 44) published in 2009 and lowest (n = 17) in 2004 (Table 1). During the decade, over half of the studies published were descriptive or case reports (53.4%, n = 151), followed by cross-sectional (20.8%, n = 59), case-control (8.5%, n = 24), cohort (6.0%, n = 17) and ecological (4.2%, n = 12) (Table 1). In 2011 and 2012, descriptive and cross-sectional studies presented almost an equal number of publications. Study design was not explicitly stated in 46.6% (n = 188) of publications; additionally, 24 (17.5%) incorrectly stated study design based on an evaluation of the information provided (Figure 2). Comparisons groups were utilized in only 39.6% (n = 112) of the publications, and only 13.4% (n = 38) included non-infected subjects (“healthy controls”) as their comparison group (Figure 3).

**Table 1:** Number of publications per year by study design of published epidemiological observational research on leishmaniasis in Brazil, 2002–2012.

Publication Year	Full Text not Available	Descriptive	Case Report	Ecological	Cross-sectional	Case-control	Cohort	Total
2002	2	8	3	4	4	2	2	25 (8.80%)
2003	6	7	5	0	0	1	1	20 (7.90%)
2004	1	7	3	1	2	2	1	17 (6.00%)
2005	4	4	3	0	6	3	1	21 (7.45%)
2006	1	13	3	0	5	0	2	24 (8.50%)
2007	3	12	6	0	7	3	0	31 (11.00%)
2008	0	8	4	2	3	1	2	20 (7.10%)
2009	2	25	3	1	10	3	0	44 (15.60%)
2010	0	8	4	2	3	5	2	24 (8.50%)
2011	0	11	2	2	12	0	2	29 (10.30%)
2012	0	6	6	0	7	4	4	27 (9.50%)
Total	21 (7.45%)	109 (38.50%)	42 (14.80%)	12 (4.20%)	59 (20.80%)	24 (8.40%)	17 (6.00%)	283 (100%)

**Figure 4:**
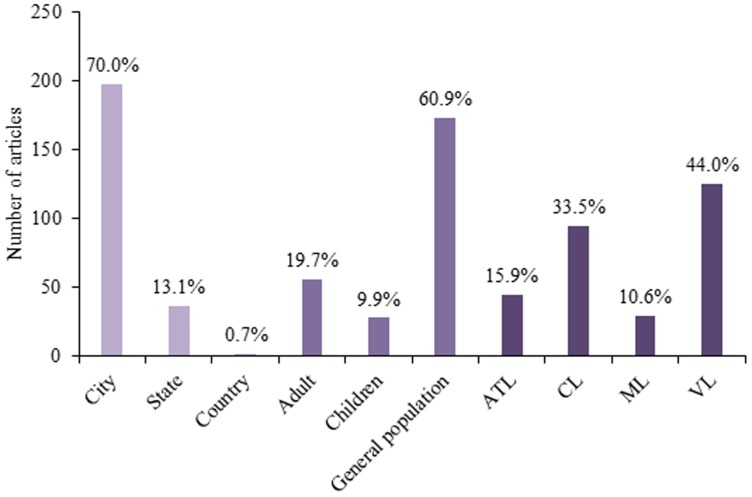
Location, group of interest and clinical forms of published epidemiological observational research on leishmaniasis in Brazil, 2002–2012. ATL  =  American tegumentary leishmaniasis; CL  =  cutaneous leishmaniasis; ML  =  mucocutaneous leishmaniasis; VL  =  visceral leishmaniasis.

Of the total publications, 188 (66.4%) studies provided a full text in English. Free full text was available for 67.1% (n = 190) of the studies. Of these, 37.9% (n = 72) were available only in Portuguese (Figure 3). The large majority of studies were performed at the city-level (62.9%, n = 178), in contrast with only two (0.7%) studies performed at the national-level (Figure 4).

Most studies were performed among the general population (adults and children together) (60.9%) rather than a specific age group, with the main clinical form of interest being visceral leishmaniasis (VL) (44.0%), followed by cutaneous leishmaniasis (CL) (33.5%), American tegumentary leishmaniasis (ATL) (15.9%) and mucosal leishmaniasis (ML) (10.6%) (Figure 4).

**Figure 5:**
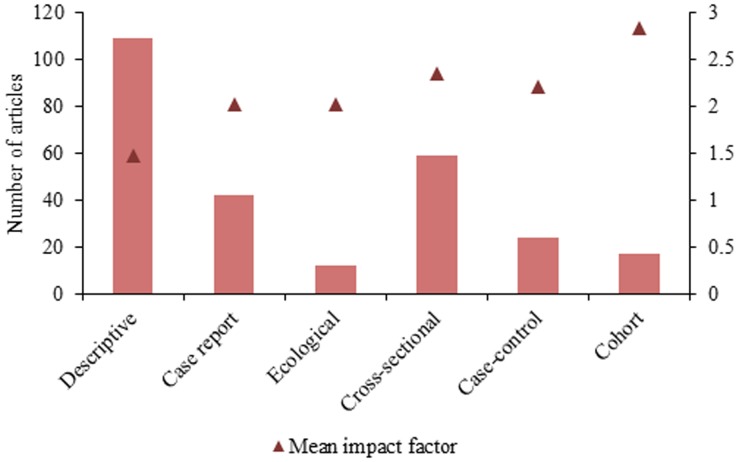
Study design and mean journal impact factor of published epidemiological observational research on leishmaniasis in Brazil, 2002–2012.

During the time period, total number of citations resulting from articles published in English was 2,246 and 499 for articles available in Portuguese only. The average number of citations produced by papers published in English (12.0 citations per article) was nearly double that produced by papers with Portuguese text only version (6.1 citations per article). Six of the top ten most published journals were Brazilian journals. More than half (58.45%, n = 165) of the papers were published in these ten journals, with 20.8% of all articles originating from one journal alone (Table 2). The mean impact factor (MIF) of these ten journals was 1.97 (SD = 1.61) (Table 2). The MIF of all publications was 1.90 (SD = 1.62), which was observed to increase with higher evidence study designs (Figure 5). Cohort studies represented the highest average MIF publications (2.83, SD = 1.55) and descriptive studies the lowest (1.47, SD = 1.00). MIF of publications on ML (2.63, SD = 1.65) was highest, followed by VL (2.02, SD = 1.85), CL (1.83, SD = 1.45), and ATL (1.64, SD = 1.21). Only 6% (n = 17) of the studies were published in journals with an impact factor higher than 4.0 (Table 3).

**Table 2:** Top ten journals according to number of publications and journal impact factor for published epidemiological observational research on leishmaniasis in Brazil, 2002–2012.

#	Journal Ranked by Number of Articles	Frequency	Percent	2012 JCR Impact Factor
1.	Revista da Sociedade Brasileira de Medicina Tropical	59	20.75%	0.97
2.	The American Journal of Tropical Medicine and Hygiene	27	9.50%	2.90
3.	Cadernos de Saúde Pública	20	7.05%	1.29
4.	Transactions of the Royal Society of Tropical Medicine and Hygiene	15	5.30%	2.46
5.	Anais Brasileiros de Dermatologia	11	3.85%	0.62
6.	Acta Tropica	10	3.50%	2.51
7.	Revista do Instituto de Medicina Tropical de São Paulo	7	2.45%	0.96
8.	International Journal of Dermatology	6	2.10%	1.36
9.	Revista Brasileira de Epidemiologia	6	2.10%	0.70
10.	Journal of Infectious Diseases	5	1.75%	5.91

**Table 3:** Top ten journals according to impact factor and number of publications for published epidemiological observational research on leishmaniasis in Brazil, 2002–2012.

#	Journal Ranked by Number of Impact Factor	2012 JCR Impact Factor	Frequency
1.	Journal of Clinical Oncology	17.26	1
2.	Clinical Infectious Diseases	8.98	1
3.	Kidney International	6.97	1
4.	Emerging Infectious Diseases	6.31	1
5.	Journal of Infectious Diseases	5.91	5
6.	PLoS Neglected Tropical Diseases	4.96	5
7.	Journal of the American Geriatrics Society	4.63	1
8.	The British Journal of Dermatology	4.16	1
9.	Infection and Immunity	4.06	1
10.	The Journal of Infection	3.77	1

**Figure 6:**
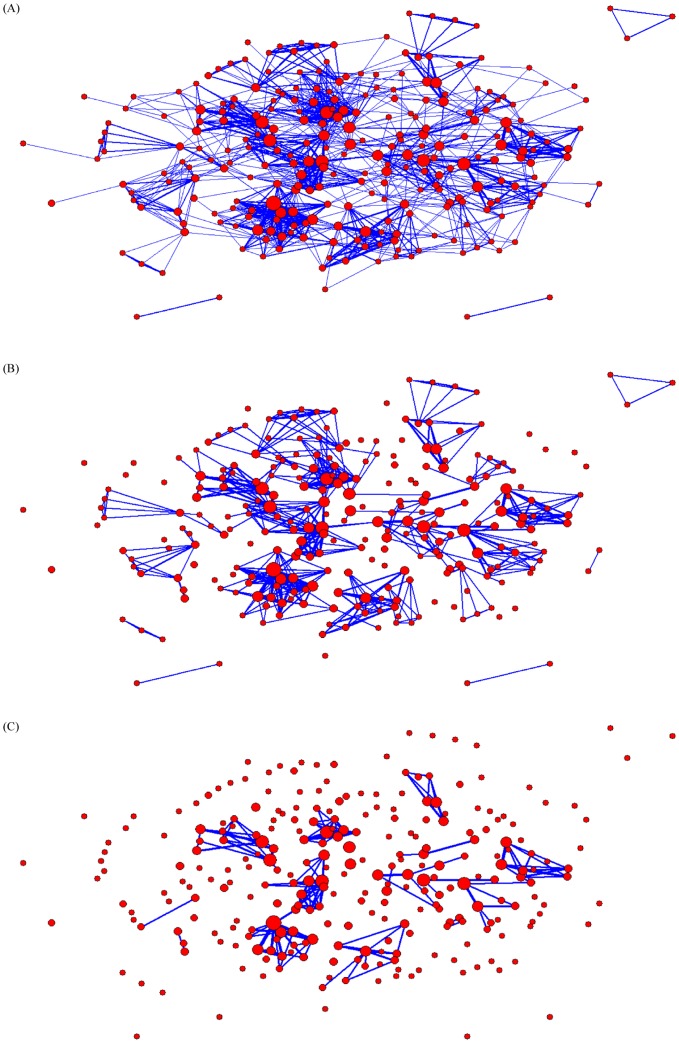
Coauthorship network of authors with at least two publications during the study period for (A) at least one or more collaborations, (B) at least two or more collaborations, and (C) at least three or more collaborations. Red circles represent coauthors, and the size of the circle represents the number of publications they published during the study period. Blue lines represent collaborations on publication (shared coauthorship on a paper), and the thickness of the line represents number of collaborations.

A total of 929 coauthors were identified among 210 publications available in the Web of Science database for bibliometric analysis. Of these, 70.9% (n = 659) published only one article as a coauthor during the ten-year study period. Coauthorship networks of authors with at least two publications revealed the number of authors who participate in ongoing collaborations rapidly decreased after just three collaborations (Figure 6). None of the top ten producing authors for observational epidemiological studies in Brazil were affiliate with a School of Public Health or Epidemiology Department.

## Discussion

In the present study, epidemiologic studies incorporating human subjects were observed to represent only a small fraction of studies on leishmaniasis in Brazil during the recent ten-year study period. Descriptive studies and case reports are widely considered to be surveillance rather than observational epidemiological research since they do not assess a hypothesis or include a comparison group. Thus, excluding these two study types, the number becomes even more disparate, with only 6.6% of studies being observational epidemiology study designs capable of producing some level of evidence for the literature. With the exception of 2009, production in the area of epidemiology remained relatively stagnant during the study period. While 2009 saw a peak in publications, nearly double the normal production, this increase was mainly due to a disproportionate increase in descriptive studies that year. The main research focus among the body of leishmaniasis research was on non-human subject research involving the pathogen, vectors and/or reservoirs. While this is an important area for developing control methods, not including human subjects greatly hinders the scaling-up process [19]. Evaluating the quality of these studies was outside the scope of the present work, but most studies of this type were observed to mirror that in the human population as being mainly descriptive studies that reported seropositive canines or infected vectors in a specific location. The second most common research focus was at the molecular level. The abundance of molecular studies versus clinical trials or *in-vitro* drug/vaccine testing (24.5% vs. 8.7%) also suggested a possible gap in scaling up of treatment as well as molecular epidemiological techniques. Given the complexity of the parasite transmission cycle and potential for clonal and sexual reproduction of *Leishmania* spp., arriving at a consensus among experts as to the molecular typing method of choice remains a challenge [20]. However, with several methods of varying levels of discriminatory power available, epidemiologists presently have a working molecular toolbox and are able select the best method based on the epidemiological design of the study by assessing the discriminatory power required to distinguish meaningful clinical/epidemiological differences in the human population. Like with other epidemiological research, molecular epidemiological research requires a representative sample and study design that incorporates a comparison group, which was rarely observed here.

In the overall body of leishmaniasis epidemiological research in Brazil, over one third of studies did not incorporate a comparison group, as reflected in the abundance of purely descriptive studies, and an even smaller fraction (around one eighth) of studies included non-infected or “healthy” control group(s). Upon closer inspection, the majority (55.3%, n = 22/38) of studies which used non-infected controls were cross-sectional studies. This means that for most of these studies healthy controls were not specifically sampled for comparison with cases, but rather were part of a prevalence study. Studies without comparison groups do not allow the determination of causes, associations or risk factors for the disease [21]. Descriptive studies, and even cross-sectional studies, do not produce evidence that can be used to justify population interventions and normally only lead to more studies being needed to generate workable hypotheses [21]. While important as pilot studies, the differentiation between surveillance and epidemiological research should be made clear during the study design phase. Moreover, studies performed among populations from a single city represented more than 70% of publications, resulting in a localized view of the problem and possibly lacking in applicability to different regions of Brazil given its continental size and varying biomes. Leishmaniasis is included as a notifiable disease in the Brazilian National Notifiable Disease Surveillance System [22]. This system could function as the basis for a national prospective study to assess novel areas for population-based interventions, unifying many of the city-by-city descriptive studies and allowing for a larger more concerted study with regional comparisons.

The higher number of cross-sectional vs. descriptive studies, albeit just by one study, and the highest number of cohort and case-control studies occurring in the last two years of the study period suggest progress is being made in the area. Also encouraging is the increase from 24.0% in 2002 to 40.7% in 2012 in the number of studies which stated study design in the text. However, it should also be noted that the number of studies which incorrectly stated the study design also increased 4.0% to 14.8% during the same period. Only four of the 17 studies identified as cohort studies provided and/or correctly stated study design in the text, casting doubt on the quality and design of these studies which are intended to represent the highest of evidence among observational studies.

Among the literature from Brazil analyzed here, nearly 35% of articles were available in Portuguese text only. Furthermore, we found articles with English versions available had over twice as many citations on average than Portuguese-only articles. As English is currently accepted as the universal language for scientific publication, over one third of articles on leishmaniasis were restricted to a smaller portion of the scientific community, creating a barrier for collaboration, constructive criticism and discussion of the work with the general international community [23], [24]. Interaction with peers is an imperative part of the scientific method as well as important for sharing successful policies and novel strategies around the globe [25]. The reduction in the percentage of Portuguese-only text from 20–55% from 2002 to 2009 to 7–8% from 2010 to 2012 is a hopeful sign that the availability of English texts is improving. This may also reflect an increase in the number of Brazilian journals requiring English texts, including the most published journal for leishmaniasis in Brazil, Revista da Sociedade Brasileira de Medicina Tropical, which changed to English as its official publication language in 2012.

While impact factors are controversial as a method for evaluating journal quality [26], we were able to use them here to reflect trends in publication quality among articles on leishmaniasis in Brazil. As expected, higher quality evidence study designs, mainly cohort and case-control designs, resulted in publications in journals with higher impact factors. This is reflected in the generalizability of the findings of these types of studies in comparison to descriptive or prevalence studies. While descriptive and prevalence studies may be useful for reporting the presence of *Leishmania* species or newly endemic areas, they often do not provide results that may be useful to other researchers in other regions and/or countries, such as associations or risk factors. Moreover, as over 70% of authors only published one study as a coauthor during the study period, many of these descriptive studies or pilot studies likely did not result in continued hypothesis building and generation of new research questions. Coauthor networks revealed researchers are also not maintaining collaborations and consistent research groups in the area. Epidemiology functions as a three-part cycle, beginning by identifying trends and outbreaks through surveillance, designing epidemiological observational studies to assess our hypotheses on these trends, and then creating and instating interventions which leads to continued surveillance to monitor the effect of interventions. Therefore, this abundance of descriptive studies and discontinuation of follow-up publications and collaborations makes the epidemiological cycle top heavy by focusing on surveillance and not following through to conduct observational research. Two potential reasons for this phenomenon may be the emphasis on scientific production by Brazilian governmental institutions responsible for the evaluation of graduate programs [27] as well as a lack of opportunities for young researchers in the field of NTDs to build a strong well-funded research lab, attract students and publish in top international journals [28].

Although the level of evidence produced by the articles remains unsatisfactory to develop targeted interventions, results in the present study from the most recent years did suggest an improvement in research quality in the area. In resource-limited settings, as found in Brazil, there is a need to optimize financial resources into projects with relevance and objectiveness [29]. Thus, with a predominance of studies that generate low-level of evidence, the cost-effectiveness of these publications must be considered as a driving factor in improving the quality of targeted observational research. Based on the findings here, we propose eight recommendations for researchers working in the area of leishmaniasis epidemiology as a basis for improving their current and future studies (Table 4). Mastering the basics of epidemiology, designing efficient and effective hypothesis-driven observational studies and applying the results to create novel interventions by researchers in the area could be the next significant advancement in leishmaniasis prevention and control.

**Table 4:** Recommendations for improving the quality of observational epidemiological research on leishmaniasis based on a review of published research in Brazil (2002–2012).

1.	Biologist and medical professionals are encouraged to consult a trained epidemiologist and the STROBE checklist before initiating a project.
2.	Epidemiological study design courses should be stimulated among neglected tropical disease conferences to set higher standards for future research.
3.	Journals, both national and international, should discourage articles that are purely descriptive if they do not provide new evidence for the literature base.
4.	The STROBE checklist should be incorporated into medical school and science department curriculums as well as required for publication in both international and Brazilian journals in the area.
5.	Using the term “cohort” study to refer to research conducted among a group of patients/subjects, rather than a cohort study that follows exposed and unexposed populations over time, should be discouraged during the review process.
6.	In many cases, smaller studies with a representative sample and comparison group(s) can provide stronger evidence than larger descriptive/prevalence studies. In addition to improving statistical power and evidence, sample size calculation based on a defined research question can avoid unnecessary data and sample collection and inefficient use of resources.
7.	A standardized questionnaire for field studies in leishmaniasis needs to be developed and validated such that studies conducted in different study sites can be directly compared.
8.	Researchers with a focus on molecular epidemiological studies should put an increased emphasis on the epidemiological design of their studies, particularly in regards to collecting a representative sample of the target population; a molecular epidemiology study is only as strong as its epidemiological methods.

## Supporting Information

Text S1 List of references for articles included in the evaluation of epidemiological observational research on leishmaniasis in Brazil, 2002–2012.
(DOCX)
