# Supplementary material for: Low Quality Evidence of Epidemiological Observational Studies on Leishmaniasis in Brazil
Source: PLoS One. 2014 Sep 8;9(9):e106635. doi: 10.1371/journal.pone.0106635 (PMC4157786; doi:10.1371/journal.pone.0106635)
Supplement: Text S1 — List of references for articles included in the evaluation of epidemiological observational research on leishmaniasis in Brazil, 2002–2012. (DOCX) [file pone.0106635.s001.docx]

**Text S1**

1. Ramasawmy R, Menezes E, Magalhaes A, Oliveira J, Castellucci L, et al. (2010) The -2518bp promoter polymorphism at CCL2/MCP1 influences susceptibility to mucosal but not localized cutaneous leishmaniasis in Brazil. Infect Genet Evol 10: 607-613.

2. Antonelli LR, Dutra WO, Almeida RP, Bacellar O, Carvalho EM, et al. (2005) Activated inflammatory T cells correlate with lesion size in human cutaneous leishmaniasis. Immunol Lett 101: 226-230.

3. Liborio AB, Rocha NA, Oliveira MJ, Franco LF, Aguiar GB, et al. (2012) Acute kidney injury in children with visceral leishmaniasis. Pediatr Infect Dis J 31: 451-454.

4. Lambertucci JR, França BM, Queiroz EdM (2004) Acute pancreatitis caused by meglumine antimoniate given for the treatment of visceral leishmaniasis. Revista da Sociedade Brasileira de Medicina Tropical 37: 74-75.

5. Shaw JJ, De Faria DL, Basano SA, Corbett CE, Rodrigues CJ, et al. (2007) The aetiological agents of American cutaneous leishmaniasis in the municipality of Monte Negro, Rondonia state, western Amazonia, Brazil. Ann Trop Med Parasitol 101: 681-688.

6. Arraes SM, Veit RT, Bernal MV, Becker TC, Nanni MR (2008) [American cutaneous leishmaniasis in municipalities in the northwestern region of Parana State: use of remote sensing for analysis of vegetation types and places with disease occurrence]. Rev Soc Bras Med Trop 41: 642-647.

7. Bafica A, Oliveira F, Freitas LA, Nascimento EG, Barral A (2003) American cutaneous leishmaniasis unresponsive to antimonial drugs: successful treatment using combination of N-methilglucamine antimoniate plus pentoxifylline. Int J Dermatol 42: 203-207.

8. Murback ND, Hans Filho G, Nascimento RA, Nakazato KR, Dorval ME (2011) American cutaneous leishmaniasis: clinical, epidemiological and laboratory studies conducted at a university teaching hospital in Campo Grande, Mato Grosso do Sul, Brazil. An Bras Dermatol 86: 55-63.

9. Yarzon RM, Dorval ME, de Freitas HG, Oshiro ET (2003) [American leishmaniasis in Mato Grosso do Sul, Brazil]. Rev Soc Bras Med Trop 36 Suppl 2: 41-42.

10. Duarte JL (2003) [American leishmaniasis in Mato Grosso State, Brazil - 2002]. Rev Soc Bras Med Trop 36 Suppl 2: 44-45.

11. de Camargo-Neves VL, Brasil MT (2003) [American leishmaniasis in the state of Sao Paulo: epidemiological status in 2001-2002]. Rev Soc Bras Med Trop 36 Suppl 2: 30-35.

12. Guerra JA, Coelho LI, Pereira FR, Siqueira AM, Ribeiro RL, et al. (2011) American tegumentary leishmaniasis and HIV-AIDS association in a tertiary care center in the Brazilian Amazon. Am J Trop Med Hyg 85: 524-527.

13. Andrade MS, Brito ME, Silva ST, Lima BS, Almeida EL, et al. (2005) [American tegumentary leishmaniasis caused by *Leishmania (Viannia) braziliensis* in military training area of Zona da Mata in Pernambuco]. Rev Soc Bras Med Trop 38: 229-233.

14. Guerra JA, Barbosa M, Loureiro AC, Coelho CP, Rosa GG, et al. (2007) [American tegumentary leishmaniasis in children: epidemiological aspects of cases treated in Manaus, Amazonas, Brazil]. Cad Saude Publica 23: 2215-2223.

15. de Camargo Ferreira EVE, de Oliveira Schubach A, Valete-Rosalino CM, de Souza Coutinho R, Conceicao-Silva F, et al. (2010) American tegumentary leishmaniasis in older adults: 44 cases treated with an intermittent low-dose antimonial schedule in Rio de Janeiro, Brazil. J Am Geriatr Soc 58: 614-616.

16. Rey LC, Martins CV, Ribeiro HB, Lima AA (2005) American visceral leishmaniasis (kala-azar) in hospitalized children from an endemic area. J Pediatr (Rio J) 81: 73-78.

17. Madalosso G, Fortaleza CM, Ribeiro AF, Cruz LL, Nogueira PA, et al. (2012) American visceral leishmaniasis: factors associated with lethality in the state of sao paulo, Brazil. J Trop Med 2012: 281572.

18. Brajao de Oliveira K, Reiche EM, Kaminami Morimoto H, Pelegrinelli Fungaro MH, Estevao D, et al. (2007) Analysis of the CC chemokine receptor 5 delta32 polymorphism in a Brazilian population with cutaneous leishmaniasis. J Cutan Pathol 34: 27-32.

19. Romero GA, de la Gloria Orge Orge M, de Farias Guerra MV, Paes MG, de Oliveira Macedo V, et al. (2005) Antibody response in patients with cutaneous leishmaniasis infected by *Leishmania (Viannia) braziliensis* or *Leishmania (Viannia) guyanensis* in Brazil. Acta Trop 93: 49-56.

20. Silva LA, Romero HD, Nogueira Nascentes GA, Costa RT, Rodrigues V, et al. (2011) Antileishmania immunological tests for asymptomatic subjects living in a visceral leishmaniasis-endemic area in Brazil. Am J Trop Med Hyg 84: 261-266.

21. Viana GM, Nascimento MD, Diniz Neto JA, Rabelo EM, Binda Junior JR, et al. (2011) Anti-*Leishmania* titers and positive skin tests in patients cured of kala-azar. Braz J Med Biol Res 44: 62-65.

22. Zanoni LZ, Brustoloni YM, Melnikov P, Consolo CE (2009) Antimony containing drug and ECG abnormalities in children with visceral leishmaniasis. Biol Trace Elem Res 132: 35-40.

23. Carneiro DD, Bavia ME, Rocha WJ, Tavares AC, Cardim LL, et al. (2007) Application of spatio-temporal scan statistics for the detection of areas with increased risk for American visceral leishmaniasis in the state of Bahia, Brazil. Geospat Health 2: 113-126.

24. Caldas AJ, Costa J, Aquino D, Silva AA, Barral-Netto M, et al. (2006) Are there differences in clinical and laboratory parameters between children and adults with American visceral leishmaniasis? Acta Trop 97: 252-258.

25. Guerra JAdO, Talhari S, Paes MG, Garrido M, Talhari JM Aspectos clínicos e diagnósticos da leishmaniose tegumentar americana em militares simultaneamente expostos à infecçäo na Amazônia

Clinical and diagnostic aspects of American tegumentary leishmaniosis in soldiers simultaneously exposed to the infection in the Amazon Region. Rev Soc Bras Med Trop 36: 587-590.

26. Pedrosa CMS, Rocha EMMd Aspectos clínicos e epidemiológicos da leishmaniose visceral em menores de 15 anos procedentes de Alagoas, Brasil

Clinical and epidemiological aspects of visceral leishmaniasis in children up to 15 years of age in Alagoas, Brasil. Rev Soc Bras Med Trop 37: 300-304.

27. Caldas AdJMC, Jackson Maurício Lopes. (2002) Aspectos epidemiológicos da infecção por *Leishmania (Leishmania) Chagasi* em crianças de 0 a 5 anos do município de Raposa-MA / The epidemiological aspects of the infection by *Leishmania (Leishmania) Cahagasi*: 0-5 children in the city of Raposa-MA

Nursing (So Paulo); 5(46): 29-34, mar 2002 ilus, tab, graf

28. França EL, Mandadori MN, França JL, Botelho AdCF, Ferrari CKB, et al. Aspectos epidemiológicos da Leishmaniose Tegumentar Americana no município de Juína, Mato Grosso, Brasil

Epidemiological aspects of American Cutaneous Leishmaniasis in the city of Juína, Mato Grosso state, Brazil. Sci med 19: 103-107.

29. Secretaria de Estado da Saúde de São P Aspectos epidemiológicos da leishmaniose tegumentar americana no município de Ubatuba, litoral de São Paulo, Brasil, 1993-2003

Epidemiology of American tegumentar leishmaniasis in the municipality of Ubatuba, North coastal area of the state of São Paulo, Brazil, 1993-2003. Rev Saude Publica 38: 331-332.

30. Oliveira MRd, Maciel JN Aspectos Socioeconômicos da Leishmaniose Visceral em João Pessoa - Paraíba - Brasil

Socio-Economic Aspects of Visceral Leishmaniasis in João Pessoa - Paraíba - Brazil. Rev bras cinc sa£de 7: 63-70.

31. Borges BK, Silva JA, Haddad JP, Moreira EC, Magalhaes DF, et al. (2008) [Assessment of knowledge and preventive attitudes concerning visceral leishmaniasis in Belo Horizonte, Minas Gerais State, Brazil]. Cad Saude Publica 24: 777-784.

32. Maciel BL, Lacerda HG, Queiroz JW, Galvao J, Pontes NN, et al. (2008) Association of nutritional status with the response to infection with *Leishmania chagasi*. Am J Trop Med Hyg 79: 591-598.

33. Oliveira AL, Paniago AM, Sanches MA, Dorval ME, Oshiro ET, et al. (2008) Asymptomatic infection in family contacts of patients with human visceral leishmaniasis in Tres Lagoas, Mato Grosso do Sul State, Brazil. Cad Saude Publica 24: 2827-2833.

34. Aleixo JA, Nascimento ET, Monteiro GR, Fernandes MZ, Ramos AM, et al. (2006) Atypical American visceral leishmaniasis caused by disseminated *Leishmania amazonensis* infection presenting with hepatitis and adenopathy. Trans R Soc Trop Med Hyg 100: 79-82.

35. Carnauba D, Jr., Konishi CT, Petri V, Martinez IC, Shimizu L, et al. (2009) Atypical disseminated leishmaniasis similar to post-kala-azar dermal leishmaniasis in a Brazilian AIDS patient infected with *Leishmania (Leishmania) infantum chagasi*: a case report. Int J Infect Dis 13: e504-507.

36. Santos-Oliveira JR, Da-Cruz AM, Pires LH, Cupolillo E, Kuhls K, et al. (2011) Atypical lesions as a sign of cutaneous dissemination of visceral leishmaniasis in a human immunodeficiency virus-positive patient simultaneously infected by two viscerotropic *Leishmania* species. Am J Trop Med Hyg 85: 55-59.

37. Guimaraes LH, Machado PR, Lago EL, Morgan DJ, Schriefer A, et al. (2009) Atypical manifestations of tegumentary leishmaniasis in a transmission area of *Leishmania braziliensis* in the state of Bahia, Brazil. Trans R Soc Trop Med Hyg 103: 712-715.

38. Amato VS, Tonacio AC, Alves Mdo M (2010) An atypical presentation of cutaneous leishmaniasis. Rev Soc Bras Med Trop 43: 481.

39. Chaves RCG Avaliação epidemiológica da Leishmaniose Tegumentar Americana no estado do Amapá em 2002

Epidemiological evaluation of american leishmaniasis in Amapá State, Brazil - 2002. Rev Soc Bras Med Trop 36: 25-26.

40. Silva-Vergara ML, Silva Lde A, Maneira FR, da Silva AG, Prata A (2004) Azithromycin in the treatment of mucosal leishmaniasis. Rev Inst Med Trop Sao Paulo 46: 175-177.

41. Sangioni LA, Gebara CMdS, Aragão GM, Bezerra CAdA, Almeida CCd (2007) Busca ativa de casos de leishmaniose cutânea em humanos e cães em área periférica do município de Campo Mourão - PR, Brasil. Ciência Rural 37: 1492-1494.

42. Morejón García M, Salup Díaz RR Calazar: estudio de 18 pacientes en Brasil

Kala-azar. Study of 18 patients in Brazil. Rev cuba med gen integr 22.

43. Gomes-Silva A, de Cassia Bittar R, Dos Santos Nogueira R, Amato VS, da Silva Mattos M, et al. (2007) Can interferon-gamma and interleukin-10 balance be associated with severity of human *Leishmania (Viannia) braziliensis* infection? Clin Exp Immunol 149: 440-444.

44. Amato VS, Tuon FF, Camargo RA, Souza RM, Santos CR, et al. (2011) Can we use a lower dose of liposomal amphotericin B for the treatment of mucosal American leishmaniasis? Am J Trop Med Hyg 85: 818-819.

45. Ampuero J, Macêdo V Características clínicas da leishmaniose tegumentar em criancas de 0 a 5 anos em uma área endêmica de *Leishmania (Viannia) braziliensis*

Clinical findings of tegumentary leishmaniasis in children under five years of age in an endemic area of *Leishmania (Viannia) braziliensis*. Rev Soc Bras Med Trop 39: 22-26.

46. Xavier-Gomes LM, Costa WB, Prado PFd, Oliveira-Campos M, Leite MTdS (2009) Características clínicas e epidemiológicas da leishmaniose visceral em crianças internadas em um hospital universitário de referência no norte de Minas Gerais, Brasil. Revista Brasileira de Epidemiologia 12: 549-555.

47. Oliveira CD, Diez-Roux A, Cesar CC, Proietti FA (2006) A case-control study of microenvironmental risk factors for urban visceral leishmaniasis in a large city in Brazil, 1999-2000. Rev Panam Salud Publica 20: 369-376.

48. Vieira ML, Jacobina RR, Soares NM Casos de leishmanioses em pacientes atendidos nos Centros de Saúde e hospitais de Jacobina-BA no período de 2000 a 2004

Cases of leishmaniasis in patients treated in Health Centers and hospitals in Jacobina, Bahia, Brazil, from 2000 to 2004. Rev baiana sa£de p£blica 31: 102-114.

49. Keesen TS, Antonelli LR, Faria DR, Guimaraes LH, Bacellar O, et al. (2011) CD4(+) T cells defined by their Vbeta T cell receptor expression are associated with immunoregulatory profiles and lesion size in human leishmaniasis. Clin Exp Immunol 165: 338-351.

50. Oliveira CC, Lacerda HG, Martins DR, Barbosa JD, Monteiro GR, et al. (2004) Changing epidemiology of American cutaneous leishmaniasis (ACL) in Brazil: a disease of the urban-rural interface. Acta Trop 90: 155-162.

51. Thompson RA, Wellington de Oliveira Lima J, Maguire JH, Braud DH, Scholl DT (2002) Climatic and demographic determinants of American visceral leishmaniasis in northeastern Brazil using remote sensing technology for environmental categorization of rain and region influences on leishmaniasis. Am J Trop Med Hyg 67: 648-655.

52. Daher EF, Fonseca PP, Gerhard ES, Leitao TM, Silva Junior GB (2009) Clinical and epidemiological features of visceral leishmaniasis and HIV co-infection in fifteen patients from Brazil. J Parasitol 95: 652-655.

53. Brito ME, Silva CJ, Silva CM, Salazar PR, Coutinho JS, et al. (2008) Clinical epidemiological profile of American tegumentary leishmaniasis at the Pinto Sugar Mill in Moreno Municipality, Greater Metropolitan Recife, Pernambuco State, Brazil. Cad Saude Publica 24: 2445-2448.

54. Vieira-Goncalves R, Pirmez C, Jorge ME, Souza WJ, Oliveira MP, et al. (2008) Clinical features of cutaneous and disseminated cutaneous leishmaniasis caused by *Leishmania (Viannia) braziliensis* in Paraty, Rio de Janeiro. Int J Dermatol 47: 926-932.

55. Daher EF, Evangelista LF, Silva Junior GB, Lima RS, Aragao EB, et al. (2008) Clinical presentation and renal evaluation of human visceral leishmaniasis (kala-azar): a retrospective study of 57 patients in Brazil. Braz J Infect Dis 12: 329-332.

56. Reis Lde C, Brito ME, Almeida EL, Felix SM, Medeiros AC, et al. (2008) Clinical, epidemiological and laboratory aspects of patients with American cutaneous leishmaniasis in the State of Pernambuco. Rev Soc Bras Med Trop 41: 439-443.

57. Domingues M, Menezes Y, Ostronoff F, Calixto R, Florencio R, et al. (2009) Coexistence of Leishmaniasis and Hodgkin's lymphoma in a lymph node. J Clin Oncol 27: e184-185.

58. Sousa-Gomes MLd, Maia-Elkhoury ANS, Pelissari DM, Lima Junio FEFd, Sena JMd, et al. Coinfecção *Leishmania*-HIV no Brasil: aspectos epidemiológicos, clínicos e laboratoriais

Co-infection *Leishmania*/HIV in Brazil: epidemiological, clinical and laboratorial aspects. Epidemiol serv sa£de 20: 519-526.

59. Araujo SA, Nascentes Queiroz TC, Demas Alvares Cabral MM (2010) Colonic leishmaniasis followed by liver transplantation. Am J Trop Med Hyg 83: 209.

60. Boaventura VS, Cafe V, Costa J, Oliveira F, Bafica A, et al. (2006) Concomitant early mucosal and cutaneous leishmaniasis in Brazil. Am J Trop Med Hyg 75: 267-269.

61. Gomes CM, Giannella-Neto D, Gama ME, Pereira JC, Campos MB, et al. (2007) Correlation between the components of the insulin-like growth factor I system, nutritional status and visceral leishmaniasis. Trans R Soc Trop Med Hyg 101: 660-667.

62. Falqueto A, Ferreira AL, dos Santos CB, Porrozzi R, da Costa MV, et al. (2009) Cross-sectional and longitudinal epidemiologic surveys of human and canine *Leishmania infantum* visceral infections in an endemic rural area of southeast Brazil (Pancas, Espirito Santo). Am J Trop Med Hyg 80: 559-565.

63. Crescente JA, Silveira FT, Lainson R, Gomes CM, Laurenti MD, et al. (2009) A cross-sectional study on the clinical and immunological spectrum of human *Leishmania (L.) infantum chagasi* infection in the Brazilian Amazon region. Trans R Soc Trop Med Hyg 103: 1250-1256.

64. Gomes KW, Benevides AN, Vieira FJ, Burlamaqui MP, Vieira Mde A, et al. (2012) Cutaneous leishmaniasis in a patient with ankylosing spondylitis using adalimumab. Rev Bras Reumatol 52: 447-452.

65. Fernandes IM, Baptista MA, Barbon TR, Oliveira JF, Oliveira RC, et al. (2002) Cutaneous leishmaniasis in kidney transplant recipient. Transplant Proc 34: 504-505.

66. Tuon FF, Sabbaga Amato V, Floeter-Winter LM, de Andrade Zampieri R, Amato Neto V, et al. (2007) Cutaneous leishmaniasis reactivation 2 years after treatment caused by systemic corticosteroids - first report. Int J Dermatol 46: 628-630.

67. Castellucci L, Jamieson SE, Miller EN, Menezes E, Oliveira J, et al. (2010) CXCR1 and SLC11A1 polymorphisms affect susceptibility to cutaneous leishmaniasis in Brazil: a case-control and family-based study. BMC Med Genet 11: 10.

68. Luz KG, Tuon FF, Duarte MI, Maia GM, Matos P, et al. (2010) Cytokine expression in the duodenal mucosa of patients with visceral leishmaniasis. Rev Soc Bras Med Trop 43: 393-395.

69. Nogueira MF, Goto H, Sotto MN, Cucé LC (2008) Cytokine profile in Montenegro skin test of patients with localized cutaneous and mucocutaneous leishmaniasis. Revista do Instituto de Medicina Tropical de São Paulo 50: 333-337.

70. Stober CB, Jeronimo SM, Pontes NN, Miller EN, Blackwell JM (2012) Cytokine responses to novel antigens in a peri-urban population in Brazil exposed to *Leishmania infantum chagasi*. Am J Trop Med Hyg 87: 663-670.

71. (2004) [Data on hospital infection, external causes of death, and visceral leishmaniose in the state of Sao Paulo, Brazil]. Rev Saude Publica 38: 141-144.

72. Fagundes-Silva GA, Vieira-Goncalves R, Nepomuceno MP, de Souza MA, Favoreto S, Jr., et al. (2012) Decrease in anti-*Leishmania* IgG3 and IgG1 after cutaneous leishmaniasis lesion healing is correlated with the time of clinical cure. Parasite Immunol 34: 486-491.

73. Palatnik-de-Sousa CB, Silva-Antunes I, Morgado Ade A, Menz I, Palatnik M, et al. (2009) Decrease of the incidence of human and canine visceral leishmaniasis after dog vaccination with Leishmune in Brazilian endemic areas. Vaccine 27: 3505-3512.

74. Mulvaney P, Aram G, Maggiore PR, Kutzner H, Carlson JA (2009) Delay in diagnosis: trauma- and coinfection-related cutaneous leishmaniasis because of *Leishmania guyanensis* infection. J Cutan Pathol 36: 53-60.

75. Machado PR, Carvalho AM, Machado GU, Dantas ML, Arruda S (2011) Development of cutaneous leishmaniasis after *Leishmania* skin test. Case Rep Med 2011: 631079.

76. Moreno EC, Melo MN, Lambertucci JR, Serufo JC, Andrade AS, et al. (2006) Diagnosing human asymptomatic visceral leishmaniasis in an urban area of the State of Minas Gerais, using serological and molecular biology techniques. Rev Soc Bras Med Trop 39: 421-427.

77. Cavalcanti AT, Medeiros Z, Lopes F, Andrade LD, Ferreira Vde M, et al. (2012) Diagnosing visceral leishmaniasis and HIV/AIDS co-infection: a case series study in Pernambuco, Brazil. Rev Inst Med Trop Sao Paulo 54: 43-47.

78. Fischer M, GomesPaes M, Reinel D, Talhari S (2002) [Diffuse infiltration of the external ear in a 59-year-old Brazilian patient. "New world" cutaneous leishmaniasis (leishmaniasis tegumentar Americana)]. Hautarzt 53: 342-346.

79. Ogawa MM, Casseb Ruete L, Michalany N, Tomimori-Yamashita J (2006) Disseminated cutaneous leishmaniasis, an emerging form of cutaneous leishmaniasis: report of two cases. Int J Dermatol 45: 869-871.

80. Sousa AQ, Pompeu MM, Solon FR, Frutuoso MS, Teixeira MJ, et al. (2006) Disseminated cutaneous leishmaniasis: a patient with 749 lesions. Braz J Infect Dis 10: 230.

81. Motta AC, Arruda D, Souza CS, Foss NT (2003) Disseminated mucocutaneous leishmaniasis resulting from chronic use of corticosteroid. Int J Dermatol 42: 703-706.

82. Lima AP, Minelli L, Teodoro U, Comunello É (2002) Distribuição da leishmaniose tegumentar por imagens de sensoreamento remoto orbital, no Estado do Paraná, Brasil. Anais Brasileiros de Dermatologia 77: 681-692.

83. Aguiar V, Goncalves GMdS, Nascimento LAd, Gomes RMdA Distribuição dos casos de Leishmaniose Tegumentar Americana (LTA) em Pernambuco no ano 2002

Distribution of cases of american leishmaniasis in Pernambuco, Brazil in 2002. Rev Soc Bras Med Trop 36: 46-47.

84. Aguiar V, Goncalves GMdS, Farias FD Distribuição dos casos de Leishmaniose Visceral Humana (Calazar) em Pernambuco no ano 2002

Distribution of cases of human visceral leishmaniasis in Pernambuco, Brazil in 2002. Rev Soc Bras Med Trop 36: 39-40.

85. Cota GF, Gomes LI, Pinto BF, Santos-Oliveira JR, Da-Cruz AM, et al. (2012) Dyarrheal Syndrome in a Patient Co-Infected with *Leishmania infantum* and *Schistosoma mansoni*. Case Rep Med 2012: 240512.

86. de Araujo VE, Morais MH, Reis IA, Rabello A, Carneiro M (2012) Early clinical manifestations associated with death from visceral leishmaniasis. PLoS Negl Trop Dis 6: e1511.

87. Martins LM, Rebêlo JMM, Santos MCFVd, Costa JML, Silva ARd, et al. (2004) Ecoepidemiologia da leishmaniose tegumentar no Município de Buriticupu, Amazônia do Maranhão, Brasil, 1996 a 1998. Cadernos de Saúde Pública 20: 735-743.

88. Diniz DS, Costa AS, Escalda PM (2012) The effect of age on the frequency of adverse reactions caused by antimony in the treatment of American tegumentary leishmaniasis in Governador Valadares, State of Minas Gerais, Brazil. Rev Soc Bras Med Trop 45: 597-600.

89. Rebelo JM (2008) [El Nino episodes and temporal distribution of kala azar on Sao Luis island, Maranhao State, Brazil]. Cad Saude Publica 24: 1713-1714.

90. Nascimento ET, Moura ML, Queiroz JW, Barroso AW, Araujo AF, et al. (2011) The emergence of concurrent HIV-1/AIDS and visceral leishmaniasis in Northeast Brazil. Trans R Soc Trop Med Hyg 105: 298-300.

91. Oliveira AL, Paniago AM, Dorval ME, Oshiro ET, Leal CR, et al. (2006) [Emergent outbreak of visceral leishmaniasis in Mato Grosso do Sul State]. Rev Soc Bras Med Trop 39: 446-450.

92. Jeronimo SM, Duggal P, Braz RF, Cheng C, Monteiro GR, et al. (2004) An emerging peri-urban pattern of infection with *Leishmania chagasi*, the protozoan causing visceral leishmaniasis in northeast Brazil. Scand J Infect Dis 36: 443-449.

93. Bedoya-Pacheco SJ, Araujo-Melo MH, Valete-Rosalino CM, Pimentel MI, Conceicao-Silva F, et al. (2011) Endemic tegumentary leishmaniasis in Brazil: correlation between level of endemicity and number of cases of mucosal disease. Am J Trop Med Hyg 84: 901-905.

94. SALGADO FILHO NF, Telma Márcia A.F. e COSTA, Jackson M.L.. (2003) Envolvimento da função renal em pacientes com leishmaniose visceral (calazar). Rev Soc Bras Med Trop [online] 2003, vol36, n2, pp 217-221 ISSN 0037-8682 http://dxdoiorg/101590/S0037-86822003000200004

95. Silva NSd, Muniz VD (2009) Epidemiologia da leishmaniose tegumentar americana no Estado do Acre, Amazônia brasileira. Cadernos de Saúde Pública 25: 1325-1336.

96. Silva-Nunes Md, Cavasini CE, Silva NSd, Galati EAB (2008) Epidemiologia da Leishmaniose Tegumentar e descrição das populações de flebotomíneos no município de Acrelândia, Acre, Brasil. Revista Brasileira de Epidemiologia 11: 241-251.

97. Guerra JAdO, Ribeiro JAS, Coelho LIdARdC, Barbosa MdGV, Paes MG (2006) Epidemiologia da leishmaniose tegumentar na Comunidade São João, Manaus, Amazonas, Brasil. Cadernos de Saúde Pública 22: 2319-2327.

98. Follador I, Araujo C, Bacellar O, Araujo CB, Carvalho LP, et al. (2002) Epidemiologic and immunologic findings for the subclinical form of *Leishmania braziliensis* infection. Clin Infect Dis 34: E54-58.

99. Jirmanus L, Glesby MJ, Guimaraes LH, Lago E, Rosa ME, et al. (2012) Epidemiological and clinical changes in American tegumentary leishmaniasis in an area of *Leishmania (Viannia) braziliensis* transmission over a 20-year period. Am J Trop Med Hyg 86: 426-433.

100. Curti MC, Silveira TG, Arraes SM, Bertolini DA, Zanzarini PD, et al. (2011) Epidemiological and clinical characteristics of cutaneous leishmaniasis and their relationship with the laboratory data, south of Brazil. Braz J Infect Dis 15: 12-16.

101. de Castro EA, Soccol VT, Membrive N, Luz E (2002) [Epidemiological and clinical study of 332 cases of cutaneous leishmaniasis in the north of Parana State from 1993 to 1998]. Rev Soc Bras Med Trop 35: 445-452.

102. Nunes AG, Paula EV, Teodoro R, Prata A, Silva-Vergara ML (2006) [Epidemiological aspects of American tegumentary leishmaniasis in Varzelandia, Minas Gerais, Brazil]. Cad Saude Publica 22: 1343-1347.

103. Prado PF, Rocha MF, Sousa JF, Caldeira DI, Paz GF, et al. (2011) Epidemiological aspects of human and canine visceral leishmaniasis in Montes Claros, State of Minas Gerais, Brazil, between 2007 and 2009. Rev Soc Bras Med Trop 44: 561-566.

104. Chaves RC (2003) [Epidemiological evaluation of american leishmaniasis in Amapa State, Brazil - 2002]. Rev Soc Bras Med Trop 36 Suppl 2: 25-26.

105. de Bustamante MC, Pereira MJ, Schubach Ade O, da Fonseca AH (2009) Epidemiological profile of cutaneous leishmaniasis in an endemic region in the State of Rio de Janeiro, Brazil. Rev Bras Parasitol Vet 18: 34-40.

106. Nunes Wda S, Araujo SR, Calheiros CM (2010) Epidemiological profile of leishmaniasis at a reference service in the state of Alagoas, Brazil, from January 2000 to September 2008. Braz J Infect Dis 14: 342-345.

107. Silva AR, Tauil PL, Cavalcante MN, Medeiros MN, Pires BN, et al. (2008) [Epidemiological situation of visceral leishmaniasis on the Island of Sao Luis, State of Maranhao]. Rev Soc Bras Med Trop 41: 358-364.

108. de Oliveira DM, Pacheco ED, Araujo FP, Coelho MH (2003) [Epidemiological status of american leishmaniasis in Alagoas State, Brazil - 2002]. Rev Soc Bras Med Trop 36 Suppl 2: 23-24.

109. Gontijo CM, da Silva ES, de Fuccio MB, de Sousa MC, Pacheco RS, et al. (2002) Epidemiological studies of an outbreak of cutaneous leishmaniasis in the Rio Jequitinhonha Valley, Minas Gerais, Brazil. Acta Trop 81: 143-150.

110. (2004) [Epidemiology of American tegumentar leishmaniasis in the municipality of Ubatuba, North coastal area of the state of Sao Paulo, Brazil, 1993-2003]. Rev Saude Publica 38: 331-332.

111. Manoel ER, Martins F (2003) [Epidemiology of leishmaniasis in Goias State, Brazil - 2002]. Rev Soc Bras Med Trop 36 Suppl 2: 19-20.

112. Name RQ, Borges KT, Nogueira LSC, Sampaio JHD, Tauil PL, et al. (2005) Estudo clínico, epidemiológico e terapêutico de 402 pacientes com leishmaniose tegumentar americana atendidos no Hospital Universitário de Brasília, DF, Brasil. Anais Brasileiros de Dermatologia 80: 249-254.

113. Sampaio RNR, Gonçalves MdC, Leite VA, França BV, Santos G, et al. Estudo da transmissão da leishmaniose tegumentar americana no Distrito Federal

Study on the transmission of American cutaneous leishmaniasis in the Federal District. Rev Soc Bras Med Trop 42: 686-690.

114. Vanzeli AC, Kanamura HY Estudo de fatores socioambientais associados á ocorrência de leishmaniose tegumentar americana no município de Ubatuba, SP, Brasil

Study of social and environmental factors associatedn to the ocurrence of american tegumentary leishmaniasis in the municipality of Ubatuba, SP, Brazil. Rev panam infectol 9: 20-25.

115. Naiff Júnior RD, Pinheiro FG, Naiff MdF, Souza IdSe, Castro LM, et al. Estudo de uma série de casos de leishmaniose tegumentar americana no município de Rio Preto da Eva, Amazonas, Brasil

Case series study of american cutaneous leishmaniasis in Rio Preto da Eva municipality, Amazonas State, Brazil. Rev patol trop 38: 103-114.

116. Oliveira ÉA, Celidônio FA, Silveira TGV, Lonardoni MVC Evaluation of HIV-*Leishmania* co-infection in patients from the northwestern Paraná state, Brazil

Avaliação da co-infecção HIV-*Leishmania* em pacientes da região noroeste do estado do Paraná, Brasil. Acta sci, Health sci 33: 19-24.

117. Lima Verde FA, Lima Verde FA, Lima Verde IA, Silva Junior GB, Daher EF, et al. (2007) Evaluation of renal function in human visceral leishmaniasis (kala-azar): a prospective study on 50 patients from Brazil. J Nephrol 20: 430-436.

118. Mestre GLdC, Fontes CJF A expansão da epidemia da leishmaniose visceral no Estado de Mato Grosso, 1998-2005

The spread of the visceral leishmaniasis epidemic in the State of Mato Grosso, 1998-2005. Rev Soc Bras Med Trop 40: 42-48.

119. Mendes WdS, Silva AAMd, Trovão JdR, Silva ARd, Costa JML (2002) Expansão espacial da leishmaniose visceral americana em São Luis, Maranhão, Brasil. Revista da Sociedade Brasileira de Medicina Tropical 35: 227-231.

120. Mendes DG, Lauria-Pires L, Nitz N, Lozzi SP, Nascimento RJ, et al. (2007) Exposure to mixed asymptomatic infections with *Trypanosoma cruzi*, *Leishmania braziliensis* and *Leishmania chagasi* in the human population of the greater Amazon. Trop Med Int Health 12: 629-636.

121. Gouvea MV, Werneck GL, Costa CH, de Amorim Carvalho FA (2007) Factors associated to Montenegro skin test positivity in Teresina, Brazil. Acta Trop 104: 99-107.

122. Moura GS, Santos AM, Aquino DM, Silva AA, Caldas Ade J (2012) Factors associated with asymptomatic infection in family members and neighbors of patients with visceral leishmaniasis. Cad Saude Publica 28: 2306-2314.

123. Lindoso JA, Cruz LL, Spinola RM, Fortaleza CM, Nogueira PA, et al. (2006) [Factors associated with severe visceral leishmaniasis]. Rev Soc Bras Med Trop 39 Suppl 3: 133-134.

124. Cerbino Neto J, Werneck GL, Costa CH (2009) Factors associated with the incidence of urban visceral leishmaniasis: an ecological study in Teresina, Piaui State, Brazil. Cad Saude Publica 25: 1543-1551.

125. Silva AF, Latorre Mdo R, Galati EA (2010) [Factors relating to occurrences of cutaneous leishmaniasis in the Ribeira Valley]. Rev Soc Bras Med Trop 43: 46-51.

126. Castellucci L, Cheng LH, Araujo C, Guimaraes LH, Lessa H, et al. (2005) Familial aggregation of mucosal leishmaniasis in northeast Brazil. Am J Trop Med Hyg 73: 69-73.

127. Viana RB, Neiva CL, Dias AF, do Rosario e Souza EJ, de Padua PM (2010) Felty's syndrome and Kala-azar: a challenge for the rheumatologist. Rev Bras Reumatol 50: 710-713.

128. Botelho AC, Natal D (2009) [First epidemiological description of visceral leishmaniasis in Campo Grande, State of Mato Grosso do Sul]. Rev Soc Bras Med Trop 42: 503-508.

129. Azeredo-Coutinho RB, Conceicao-Silva F, Schubach A, Cupolillo E, Quintella LP, et al. (2007) First report of diffuse cutaneous leishmaniasis and *Leishmania amazonensis* infection in Rio de Janeiro State, Brazil. Trans R Soc Trop Med Hyg 101: 735-737.

130. Castellucci L, Jamieson SE, Miller EN, de Almeida LF, Oliveira J, et al. (2011) FLI1 polymorphism affects susceptibility to cutaneous leishmaniasis in Brazil. Genes Immun 12: 589-594.

131. Silveira FT, Lainson R, De Souza AA, Campos MB, Carneiro LA, et al. (2010) Further evidences on a new diagnostic approach for monitoring human *Leishmania (L.) infantum chagasi* infection in Amazonian Brazil. Parasitol Res 106: 377-386.

132. Alonso DP, Ferreira AF, Ribolla PE, de Miranda Santos IK, do Socorro Pires e Cruz M, et al. (2007) Genotypes of the mannan-binding lectin gene and susceptibility to visceral leishmaniasis and clinical complications. J Infect Dis 195: 1212-1217.

133. Monteiro WM, Neitzke HC, Lonardoni MV, Silveira TG, Ferreira ME, et al. (2008) [Geographic distribution and epidemiological features of American tegumentary leishmaniasis in old rural settlements in Parana State, Southern Brazil]. Cad Saude Publica 24: 1291-1303.

134. Dantas-Torres F, Brandao-Filho SP (2006) [Geographical expansion of visceral leishmaniasis in the State of Pernambuco]. Rev Soc Bras Med Trop 39: 352-356.

135. Palmeiro MR, Rosalino CM, Quintella LP, Morgado FN, da Costa Martins AC, et al. (2007) Gingival leishmaniasis in an HIV-negative patient. Oral Surg Oral Med Oral Pathol Oral Radiol Endod 104: e12-16.

136. Orsini M, Canela JR, Disch J, Maciel F, Greco D, et al. (2012) High frequency of asymptomatic *Leishmania* spp. infection among HIV-infected patients living in endemic areas for visceral leishmaniasis in Brazil. Trans R Soc Trop Med Hyg 106: 283-288.

137. Romero GA, Flores MR, Noronha EF, Macedo Vde O (2003) High frequency of skin reactions in patients with leishmaniasis treated with meglumine antimoniate contaminated with heavy metals: a comparative approach using historical controls. Mem Inst Oswaldo Cruz 98: 145-149.

138. Alexandrino-de-Oliveira P, Santos-Oliveira JR, Dorval ME, Da-Costa F, Pereira GR, et al. (2010) HIV/AIDS-associated visceral leishmaniasis in patients from an endemic area in Central-west Brazil. Mem Inst Oswaldo Cruz 105: 692-697.

139. Verde FA, Verde FA, Neto AS, Almeida PC, Verde EM (2011) Hormonal disturbances in visceral leishmaniasis (kala-azar). Am J Trop Med Hyg 84: 668-673.

140. Costa CH, Werneck GL, Rodrigues L, Jr., Santos MV, Araujo IB, et al. (2005) Household structure and urban services: neglected targets in the control of visceral leishmaniasis. Ann Trop Med Parasitol 99: 229-236.

141. Lonardoni MV, Silveira TG, Alves WA, Maia-Elkhoury AN, Membrive UA, et al. (2006) [Human and canine American cutaneous leishmaniasis in Mariluz, Parana State, Brazil]. Cad Saude Publica 22: 2713-2716.

142. Barao SC, de Fonseca Camargo-Neves VL, Resende MR, da Silva LJ (2007) Human asymptomatic infection in visceral leishmaniasis: a seroprevalence study in an urban area of low endemicity. Preliminary results. Am J Trop Med Hyg 77: 1051-1053.

143. Carneiro D, Bavia ME, Rocha W, Lobão J, Madureira Filho C, et al. Identificação de áreas de risco para a leishmaniose visceral americana, através de estudos epidemiológicos e sensoriamento remoto orbital, em Feira de Santana, Bahia, Brasil (2000-2002)

Identification of risk areas for visceral leishmaniasis through epidemiological studies and remote sensing data in Feira de Santana, Bahia, Brazil (2000-2002). Rev baiana sa£de p£blica 28: 19-32.

144. de Almeida AS, Medronho Rde A, Werneck GL (2011) Identification of risk areas for visceral leishmaniasis in Teresina, Piaui State, Brazil. Am J Trop Med Hyg 84: 681-687.

145. Matos GI, Covas Cde J, Bittar Rde C, Gomes-Silva A, Marques F, et al. (2007) IFNG +874T/A polymorphism is not associated with American tegumentary leishmaniasis susceptibility but can influence *Leishmania* induced IFN-gamma production. BMC Infect Dis 7: 33.

146. Castellucci L, Menezes E, Oliveira J, Magalhaes A, Guimaraes LH, et al. (2006) IL6 -174 G/C promoter polymorphism influences susceptibility to mucosal but not localized cutaneous leishmaniasis in Brazil. J Infect Dis 194: 519-527.

147. Novoa R, Bacellar O, Nascimento M, Cardoso TM, Ramasawmy R, et al. (2011) IL-17 and Regulatory Cytokines (IL-10 and IL-27) in *L. braziliensis* Infection. Parasite Immunol 33: 132-136.

148. Peruhype-Magalhaes V, Martins-Filho OA, Prata A, Silva Lde A, Rabello A, et al. (2005) Immune response in human visceral leishmaniasis: analysis of the correlation between innate immunity cytokine profile and disease outcome. Scand J Immunol 62: 487-495.

149. Sousa-Atta ML, Salame GS, D'Oliveira A, Jr., Almeida RP, Atta AM, et al. (2002) Immunoglobulin E antileishmanial antibody response in cutaneous leishmaniasis. Clin Diagn Lab Immunol 9: 101-104.

150. De Almeida Silva L, Romero HD, Prata A, Costa RT, Nascimento E, et al. (2006) Immunologic tests in patients after clinical cure of visceral leishmaniasis. Am J Trop Med Hyg 75: 739-743.

151. Almeida AF, Castro MCAB, Oliveira AP, Souza MA, Pereira VRA Immunophenotypic characterization of patients with American cutaneous leishmaniasis prior to and after treatment in Pernambuco, Brazil. J venom anim toxins incl trop dis 17: 230-234.

152. Badaro R, Lobo I, Munos A, Netto EM, Modabber F, et al. (2006) Immunotherapy for drug-refractory mucosal leishmaniasis. J Infect Dis 194: 1151-1159.

153. Franke CR, Ziller M, Staubach C, Latif M (2002) Impact of the El Nino/Southern Oscillation on visceral leishmaniasis, Brazil. Emerg Infect Dis 8: 914-917.

154. Tuon FF, Guedes F, Fernandes ER, Pagliari C, Amato VS, et al. (2009) In situ immune responses to interstitial pneumonitis in human visceral leishmaniasis. Parasite Immunol 31: 98-103.

155. Vendrame CM, Souza LD, Carvalho MD, Salgado K, Carvalho EM, et al. (2010) Insulin-like growth factor-I induced and constitutive arginase activity differs among isolates of *Leishmania* derived from patients with diverse clinical forms of *Leishmania braziliensis* infection. Trans R Soc Trop Med Hyg 104: 566-568.

156. Bacellar O, Faria D, Nascimento M, Cardoso TM, Gollob KJ, et al. (2009) Interleukin 17 production among patients with American cutaneous leishmaniasis. J Infect Dis 200: 75-78.

157. Vasconcellos Ede C, Pimentel MI, Schubach Ade O, de Oliveira Rde V, Azeredo-Coutinho RB, et al. (2012) Intralesional meglumine antimoniate for treatment of cutaneous leishmaniasis patients with contraindication to systemic therapy from Rio de Janeiro (2000 to 2006). Am J Trop Med Hyg 87: 257-260.

158. Cunha DF, Cunha SF, Nunes AG, Silva-Vergara ML (2009) Is an increased body mass index associated with a risk of cutaneous leishmaniasis? Rev Soc Bras Med Trop 42: 494-495.

159. Costa CH, Werneck GL, Costa DL, Holanda TA, Aguiar GB, et al. (2010) Is severe visceral leishmaniasis a systemic inflammatory response syndrome? A case control study. Rev Soc Bras Med Trop 43: 386-392.

160. Roselino AM, Chociay MF, Costa RS, Machado AA, Figueiredo JF (2008) L. (L.) chagasi in AIDS and visceral leishmaniasis (kala-azar) co-infection. Rev Inst Med Trop Sao Paulo 50: 251-254.

161. Hoyama E, Schellini SA, Stolf HO, Nakajima V (2006) Lacrimal excretory system sequelae in patients treated for leishmaniasis. Arq Bras Oftalmol 69: 333-337.

162. Matta NE, Nogueira RS, Franco AM, de Souza ESI, Mattos MS, et al. (2009) *Leishmania (Viannia) guyanensis* induces low immunologic responsiveness in leishmaniasis patients from an endemic area of the Brazilian Amazon Highland. Am J Trop Med Hyg 80: 339-344.

163. Lima ID, Queiroz JW, Lacerda HG, Queiroz PV, Pontes NN, et al. (2012) *Leishmania infantum chagasi* in northeastern Brazil: asymptomatic infection at the urban perimeter. Am J Trop Med Hyg 86: 99-107.

164. Felipe IM, Aquino DM, Kuppinger O, Santos MD, Rangel ME, et al. (2011) *Leishmania* infection in humans, dogs and sandflies in a visceral leishmaniasis endemic area in Maranhao, Brazil. Mem Inst Oswaldo Cruz 106: 207-211.

165. Pellicioli AC, Martins MA, Sant'ana Filho M, Rados PV, Martins MD (2012) Leishmaniasis with oral mucosa involvement. Gerodontology 29: e1168-1171.

166. Lima MVNd, Oliveira RZd, Lima APd, Cerino DA, Silveira TGV (2007) Leishmaniose cutânea com desfecho fatal durante tratamento com antimonial pentavalente. Anais Brasileiros de Dermatologia 82: 269-271.

167. Velozo D, Cabral A, Ribeiro MCM, Motta JdOCd, Costa IMC, et al. (2006) Leishmaniose mucosa fatal em criança. Anais Brasileiros de Dermatologia 81: 255-259.

168. Chagas AC, Pessoa FAC, Medeiros JFd, Py-Daniel V, Mesquita ÉC, et al. (2006) Leishmaniose Tegumentar Americana (LTA) em uma vila de exploração de minérios - Pitinga, município de Presidente Figueiredo, Amazonas, Brasil. Revista Brasileira de Epidemiologia 9: 186-192.

169. Guerra JAdO, Barbosa MdGV, Loureiro ACdSP, Coelho CP, Rosa GG, et al. (2007) Leishmaniose tegumentar americana em crianças: aspectos epidemiológicos de casos atendidos em Manaus, Amazonas, Brasil. Cadernos de Saúde Pública 23: 2215-2223.

170. Arraes SMAA, Veit RT, Bernal MVZ, Becker TCA, Nanni MR Leishmaniose tegumentar americana em municípios da região noroeste do estado do Paraná: utilização de sensoriamento remoto para análise do tipo de vegetação e os locais de ocorrência da doença

American cutaneous leishmaniasis in municipalities in the northwestern region of Paraná State: use of remote sensing for analysis of vegetation types and places with disease occurrence. Rev Soc Bras Med Trop 41: 642-647.

171. Camargo-Neves VLFd, Brasil MTLRF (2003) Leishmaniose Tegumentar Americana no estado de São Paulo: situação epidemiológica 2001 - 2002. Revista da Sociedade Brasileira de Medicina Tropical 36: 30-35.

172. Condino MLF, Galati EAB, Holcman MM, Salum MRB, Silva DCd, et al. Leishmaniose tegumentar americana no Litoral Norte Paulista, período 1993 a 2005

American cutaneous leishmaniasis on the northern coastline of the State of São Paulo, 1993 to 2005. Rev Soc Bras Med Trop 41: 635-641.

173. Guedes ACM, Carvalho MdLRd, Melo MN (2008) Leishmaniose tegumentar americana: apresentação pouco comum. Anais Brasileiros de Dermatologia 83: 445-449.

174. Fernandes NC, Morgan I, Maceira JP, Cuzzi T, Noe RAM (2004) Leishmaniose tegumentar americana: casuística hospitalar no Rio de Janeiro. Anais Brasileiros de Dermatologia 79: 431-439.

175. Murback NDN, Hans Filho G, Nascimento RAFd, Nakazato KRdO, Dorval MEMC (2011) Leishmaniose tegumentar americana: estudo clínico, epidemiológico e laboratorial realizado no Hospital Universitário de Campo Grande, Mato Grosso do Sul, Brasil. Anais Brasileiros de Dermatologia 86: 55-63.

176. Gomes KWP, Benevides AN, Vieira FJF, Burlamaqui MPdM, Vieira MdAeP, et al. Leishmaniose tegumentar em paciente com espondilite anquilosante utilizando adalimumabe

Cutaneous leishmaniasis in a patient with ankylosing spondylitis using adalimumab. Rev bras reumatol 52: 450-452.

177. Carvalho MdLRdF, Cor Jésus; Fernandes Hueb, Márcia Guedes; Afonso, Antônio Martins; Crocco Melo, Luís Carlos. (2002) Leishmaniose tegumentar no Estado do Mato Grosso (Brasil): estudo clínico, laboratorial e terapêutico / Tegumentary leishmaniasis in the State of Mato Grosso(Brazil): clinical, laboratoty and therapeutic studies. An Bras Dermatol; 77(1): 45-56, jan-fev 2002 ilus, graf.

178. Camargo-Neves VL, Spínola R, Lage L A Leishmaniose Visceral Americana no estado de São Paulo: situação epidemiológica em 2001-2002

American leishmaniasis in the state of São Paulo: epidemiological status in 2001-2002. Rev Soc Bras Med Trop 36: 27-29.

179. Figueiró Filho EA, Uehara SNO, Senefonte FRdA, Lopes AHA, Duarte G, et al. (2005) Leishmaniose visceral e gestação: relato de caso. Revista Brasileira de Ginecologia e Obstetrícia 27: 92-97.

180. Vieira ML, Jacobina RR, Soares NM Leishmaniose visceral em adolescente gestante

Visceral leishmaniasis in pregnant adolescent. Rev cinc md biol 6: 357-361.

181. Guerra JAO, Barros MLB, Fé NF, Guerra MVF, Castellon E, et al. (2004) Leishmaniose visceral entre índios no Estado de Roraima, Brasil: aspectos clínicoepidemiológicos de casos observados no período de 1989 a 1993. Revista da Sociedade Brasileira de Medicina Tropical 37: 305-311.

182. Queiroz MJA, Alves JGB, Correia JB Leishmaniose visceral: características clínico-epidemiológicas em crianças de área endêmica

Visceral leishmaniasis: clinical and epidemiological features of children in an endemic area. J Pediatr (Rio J) 80: 141-146.

183. Oliveira F, Bafica A, Rosato AB, Favali CB, Costa JM, et al. (2011) Lesion size correlates with *Leishmania* antigen-stimulated TNF-levels in human cutaneous leishmaniasis. Am J Trop Med Hyg 85: 70-73.

184. Freitas JSd, Santana RG, Melo SR Levantamento dos casos de Leishmaniose registrados no município de Jussara, Paraná, Brasil

A survey on cases of Leishmaniosis recorded at the municipal district of Jussara, Paraná, Brazil. Arq cincias sa£de UNIPAR 10: 23-27.

185. Tuon FF, Gomes-Silva A, Da-Cruz AM, Duarte MI, Neto VA, et al. (2008) Local immunological factors associated with recurrence of mucosal leishmaniasis. Clin Immunol 128: 442-446.

186. Silveira FT, Lainson R, Pereira EA, de Souza AA, Campos MB, et al. (2009) A longitudinal study on the transmission dynamics of human *Leishmania (Leishmania) infantum chagasi* infection in Amazonian Brazil, with special reference to its prevalence and incidence. Parasitol Res 104: 559-567.

187. Teixeira AC, Paes MG, Guerra Jde O, Prata A, Silva-Vergara ML (2007) Low efficacy of azithromycin to treat cutaneous leishmaniasis in Manaus, AM, Brazil. Rev Inst Med Trop Sao Paulo 49: 235-238.

188. dos Santos Marques LH, Gomes LI, da Rocha IC, da Silva TA, Oliveira E, et al. (2012) Low parasite load estimated by qPCR in a cohort of children living in urban area endemic for visceral leishmaniasis in Brazil. PLoS Negl Trop Dis 6: e1955.

189. da Silva RA, Mercado VT, Henriques Lde F, Ciaravolo RM, Wanderley DM (2012) Magnitude and trend of American Tegumentary Leishmaniasis in the State of Sao Paulo, Brazil, 1975 to 2008. Rev Bras Epidemiol 15: 617-626.

190. Borges VC, Ruiz MC, Gomes PM, Colombo AR, Silva Lde A, et al. (2003) [Montenegro intradermoreaction after the test sequential repetitions in Porteirinha, Minas Gerais State, Brazil]. Rev Soc Bras Med Trop 36: 249-251.

191. Camuset G, Remy V, Hansmann Y, Christmann D, Gomes de Albuquerque C, et al. (2007) [Mucocutaneous leishmaniasis in Brazilian Amazonia]. Med Mal Infect 37: 343-346.

192. Camargo RA, Tuon FF, Sumi DV, Gebrim EM, Imamura R, et al. (2010) Mucosal leishmaniasis and abnormalities on computed tomographic scans of paranasal sinuses. Am J Trop Med Hyg 83: 515-518.

193. Guerra JA, Prestes SR, Silveira H, Coelho LI, Gama P, et al. (2011) Mucosal Leishmaniasis caused by *Leishmania (Viannia) braziliensis* and *Leishmania (Viannia) guyanensis* in the Brazilian Amazon. PLoS Negl Trop Dis 5: e980.

194. Gaze ST, Dutra WO, Lessa M, Lessa H, Guimaraes LH, et al. (2006) Mucosal leishmaniasis patients display an activated inflammatory T-cell phenotype associated with a nonbalanced monocyte population. Scand J Immunol 63: 70-78.

195. Amato VS, Tuon FF, Imamura R, Abegao de Camargo R, Duarte MI, et al. (2009) Mucosal leishmaniasis: description of case management approaches and analysis of risk factors for treatment failure in a cohort of 140 patients in Brazil. J Eur Acad Dermatol Venereol 23: 1026-1034.

196. Amato VS, de Andrade HF, Duarte MI (2003) Mucosal leishmaniasis: in situ characterization of the host inflammatory response, before and after treatment. Acta Trop 85: 39-49.

197. Diniz LM, Duani H, Freitas CR, Figueiredo RM, Xavier CC (2010) Neurological involvement in visceral leishmaniasis: case report. Rev Soc Bras Med Trop 43: 743-745.

198. Soccol VT, de Castro EA, Schnell e Schuhli G, de Carvalho Y, Marques E, et al. (2009) A new focus of cutaneous leishmaniasis in the central area of Parana State, southern Brazil. Acta Trop 111: 308-315.

199. Andrade MS, Brito ME, Silva ST, Ishikawa E, Carvalho SM, et al. (2009) [New outbreak of American tegumentary leishmaniasis in a military training center in the Zona da Mata region, in the north of the State of Pernambuco]. Rev Soc Bras Med Trop 42: 594-596.

200. Costa JML, Garcia AM, Rêbelo JMM, Guimarães KM, Guimarães RM, et al. (2003) Óbito durante tratamento da leishmaniose tegumentar americana com stibogluconato de sódio bp 88® (shandong xinhua). Revista da Sociedade Brasileira de Medicina Tropical 36: 295-298.

201. Felinto de Brito ME, Andrade MS, de Almeida EL, Medeiros AC, Werkhauser RP, et al. (2012) Occupationally acquired american cutaneous leishmaniasis. Case Rep Dermatol Med 2012: 279517.

202. Dorval ME, Oshiro ET, Cupollilo E, Castro AC, Alves TP (2006) [Occurrence of American tegumentary leishmaniasis in the Mato Grosso do Sul State associated to the infection for *Leishmania (Leishmania) amazonensis*]. Rev Soc Bras Med Trop 39: 43-46.

203. Motta AC, Lopes MA, Ito FA, Carlos-Bregni R, de Almeida OP, et al. (2007) Oral leishmaniasis: a clinicopathological study of 11 cases. Oral Dis 13: 335-340.

204. Silveira FT, Ishikawa EA, De Souza AA, Lainson R (2002) An outbreak of cutaneous leishmaniasis among soldiers in Belem, Para State, Brazil, caused by *Leishmania (Viannia) lindenbergi n.* sp. A new leishmanial parasite of man in the Amazon region. Parasite 9: 43-50.

205. Moitinho LMN, Freitas LARd, Marback EF, Marback RL (2009) Papel da imunoistoquímica no diagnóstico das alterações oculares na leishmaniose tegumentar americana: relato clínico-patológico de cinco casos. Revista Brasileira de Oftalmologia 68: 152-155.

206. Coutinho SG, Pirmez C, Da-Cruz AM (2002) Parasitological and immunological follow-up of American tegumentary leishmaniasis patients. Trans R Soc Trop Med Hyg 96 Suppl 1: S173-178.

207. Gomes CM, Morais OO, Leite AS, Soares KA, Motta Jde O, et al. (2012) Periungual leishmaniasis. An Bras Dermatol 87: 148-149.

208. Monteiro WM, Neitzke HC, Silveira TG, Lonardoni MV, Teodoro U, et al. (2009) [Poles of American tegumentary leishmaniasis production in northern Parana State, Brazil]. Cad Saude Publica 25: 1083-1092.

209. Monteiro WM, Neitzke-Abreu HC, Ferreira ME, Melo GC, Barbosa M, et al. (2009) [Population mobility and production of American tegumentary leishmaniasis in the State of Parana, southern Brazil]. Rev Soc Bras Med Trop 42: 509-514.

210. Rodrigues AM, Hueb M, Nery AF, Fontes CJ (2007) Possible cardioprotective effect of angiotensin-converting enzyme inhibitors during treatment of American tegumentary leishmaniasis with meglumine antimoniate. Acta Trop 102: 113-118.

211. Bittencourt A, Silva N, Straatmann A, Nunes VL, Follador I, et al. (2003) Post-kala-azar dermal leishmaniasis associated with AIDS. Braz J Infect Dis 7: 229-233.

212. Machado de Assis TS, Rabello A, Werneck GL (2012) Predictive models for the diagnostic of human visceral leishmaniasis in Brazil. PLoS Negl Trop Dis 6: e1542.

213. Santos MA, Marques RC, Farias CA, Vasconcelos DM, Stewart JM, et al. (2002) Predictors of an unsatisfactory response to pentavalent antimony in the treatment of American visceral leishmaniasis. Rev Soc Bras Med Trop 35: 629-633.

214. Souza RM, de Oliveira IB, Paiva VC, Lima KC, dos Santos RP, et al. (2009) Presence of antibodies against *Leishmania chagasi* in haemodialysed patients. Trans R Soc Trop Med Hyg 103: 749-751.

215. Nascimento Mdo D, Souza EC, da Silva LM, Leal Pda C, Cantanhede Kde L, et al. (2005) [Prevalence of infection by *Leishmania chagasi* using ELISA (rK39 and CRUDE) and the Montenegro skin test in an endemic leishmaniasis area of Maranhao, Brazil]. Cad Saude Publica 21: 1801-1807.

216. Carranza-Tamayo CO, de Assis TS, Neri AT, Cupolillo E, Rabello A, et al. (2009) Prevalence of *Leishmania* infection in adult HIV/AIDS patients treated in a tertiary-level care center in Brasilia, Federal District, Brazil. Trans R Soc Trop Med Hyg 103: 743-748.

217. Urias EVR, Carvalho SFG, Oliveira CL, Carvalho MdLM, Teles LF, et al. (2009) Prevalência de adultos infectados por *Leishmania leishmania chagasi* entre doadores de sangue do Hemocentro Regional de Montes Claros, Minas Gerais, Brasil. Revista Brasileira de Hematologia e Hemoterapia 31: 348-354.

218. Botelho ACA, Natal D Primeira descrição epidemiológica da leishmaniose visceral em Campo Grande, Estado de Mato Grosso do Sul

First epidemiological description of visceral leishmaniasis in Campo Grande, State of Mato Grosso do Sul. Rev Soc Bras Med Trop 42: 503-508.

219. Kawa H, Sabroza PC, Oliveira RM, Barcellos C (2010) [Production of transmission foci for cutaneous leishmaniasis: the case of Pau da Fome, Rio de Janeiro, Brazil]. Cad Saude Publica 26: 1495-1507.

220. Brazuna JC, Silva EA, Brazuna JM, Domingos IH, Chaves N, et al. (2012) Profile and geographic distribution of reported cases of visceral leishmaniasis in Campo Grande, State of Mato Grosso do Sul, Brazil, from 2002 to 2009. Rev Soc Bras Med Trop 45: 601-606.

221. Werneck GL, Batista MS, Gomes JR, Costa DL, Costa CH (2003) Prognostic factors for death from visceral leishmaniasis in Teresina, Brazil. Infection 31: 174-177.

222. Silveira FT, Lainson R, Crescente JA, de Souza AA, Campos MB, et al. (2010) A prospective study on the dynamics of the clinical and immunological evolution of human *Leishmania (L.) infantum chagasi* infection in the Brazilian Amazon region. Trans R Soc Trop Med Hyg 104: 529-535.

223. Romero GA, Ishikawa E, Cupolillo E, Toaldo CB, Guerra MV, et al. (2002) The rarity of infection with *Leishmania (Viannia) braziliensis* among patients from the Manaus region of Amazonas state, Brazil, who have cutaneous leishmaniasis. Ann Trop Med Parasitol 96: 131-136.

224. Nunes CM, Pires MM, da Silva KM, Assis FD, Goncalves Filho J, et al. (2010) Relationship between dog culling and incidence of human visceral leishmaniasis in an endemic area. Vet Parasitol 170: 131-133.

225. Viana GM, Nascimento Mdo D, Rabelo EM, Diniz Neto JA, Binda Junior JR, et al. (2011) Relationship between rainfall and temperature: observations on the cases of visceral leishmaniasis in Sao Luis Island, State of Maranhao, Brazil. Rev Soc Bras Med Trop 44: 722-724.

226. Bavia ME, Carneiro DD, Gurgel Hda C, Madureira Filho C, Barbosa MG (2005) Remote Sensing and Geographic Information Systems and risk of American visceral leishmaniasis in Bahia, Brazil. Parassitologia 47: 165-169.

227. Oliveira RA, Lima CG, Mota RM, Martins AM, Sanches TR, et al. (2012) Renal function evaluation in patients with American cutaneous leishmaniasis after specific treatment with pentavalent antimonial. BMC Nephrol 13: 44.

228. Daher EF, Rocha NA, Oliveira MJ, Franco LF, Oliveira JL, et al. (2011) Renal function improvement with pentavalent antimonial agents in patients with visceral leishmaniasis. Am J Nephrol 33: 332-336.

229. Agenor Araujo Lima Verde F, Araujo Lima Verde F, De Francesco Daher E, Martins Dos Santos G, Saboia Neto A, et al. (2009) Renal tubular dysfunction in human visceral leishmaniasis (Kala-azar). Clin Nephrol 71: 492-500.

230. Oliveira RA, Diniz LF, Teotonio LO, Lima CG, Mota RM, et al. (2011) Renal tubular dysfunction in patients with American cutaneous leishmaniasis. Kidney Int 80: 1099-1106.

231. Schubach Ade O, Marzochi KB, Moreira JS, Schubach TM, Araujo ML, et al. (2005) Retrospective study of 151 patients with cutaneous leishmaniasis treated with meglumine antimoniate. Rev Soc Bras Med Trop 38: 213-217.

232. Caldas AJ, Costa JM, Silva AA, Vinhas V, Barral A (2002) Risk factors associated with asymptomatic infection by *Leishmania chagasi* in north-east Brazil. Trans R Soc Trop Med Hyg 96: 21-28.

233. Araujo-Melo MH, Meneses AM, Schubach AO, Moreira JS, Conceicao-Silva F, et al. (2010) Risk factors associated with dizziness during treatment of mucosal leishmaniasis with meglumine antimoniate: 16-year retrospective study of cases from Rio de Janeiro, Brazil. J Laryngol Otol 124: 1056-1060.

234. Oliveira MJ, Silva Junior GB, Abreu KL, Rocha NA, Garcia AV, et al. (2010) Risk factors for acute kidney injury in visceral leishmaniasis (Kala-Azar). Am J Trop Med Hyg 82: 449-453.

235. Ampuero J, Urdaneta M, Macedo Vde O (2005) [Risk factors for cutaneous leishmaniasis transmission in children aged 0 to 5 years in an endemic area of *Leishmania (Viannia) braziliensis*]. Cad Saude Publica 21: 161-170.

236. Sampaio MJ, Cavalcanti NV, Alves JG, Filho MJ, Correia JB (2010) Risk factors for death in children with visceral leishmaniasis. PLoS Negl Trop Dis 4: e877.

237. Ponte CB, Souza NC, Cavalcante MN, Barral AM, Aquino DM, et al. (2011) Risk factors for *Leishmania chagasi* infection in an endemic area in Raposa, State of Maranhao, Brazil. Rev Soc Bras Med Trop 44: 712-721.

238. Moreno EC, Melo MN, Genaro O, Lambertucci JR, Serufo JC, et al. (2005) Risk factors for *Leishmania chagasi* infection in an urban area of Minas Gerais State. Rev Soc Bras Med Trop 38: 456-463.

239. Machado-Coelho GL, Caiaffa WT, Genaro O, Magalhaes PA, Mayrink W (2005) Risk factors for mucosal manifestation of American cutaneous leishmaniasis. Trans R Soc Trop Med Hyg 99: 55-61.

240. Góes MAdO, Melo CMd, Jeraldo VdLS (2012) Série temporal da leishmaniose visceral em Aracaju, estado de Sergipe, Brasil (1999 a 2008): aspectos humanos e caninos. Revista Brasileira de Epidemiologia 15: 298-307.

241. Gama ME, Costa JM, Pereira JC, Gomes CM, Corbett CE (2004) Serum cytokine profile in the subclinical form of visceral leishmaniasis. Braz J Med Biol Res 37: 129-136.

242. Cella W, Melo SC, Dell Agnolo CM, Pelloso SM, Silveira TG, et al. (2012) Seventeen years of American cutaneous leishmaniasis in a Southern Brazilian municipality. Rev Inst Med Trop Sao Paulo 54: 215-218.

243. Oliveira AL, Brustoloni YM, Fernandes TD, Dorval ME, Cunha RV, et al. (2009) Severe adverse reactions to meglumine antimoniate in the treatment of visceral leishmaniasis: a report of 13 cases in the southwestern region of Brazil. Trop Doct 39: 180-182.

244. Morgado FN, Schubach A, Vasconcellos E, Azeredo-Coutinho RB, Valete-Rosalino CM, et al. (2010) Signs of an in situ inflammatory reaction in scars of human American tegumentary leishmaniasis. Parasite Immunol 32: 285-295.

245. Souza GF, Biscione F, Greco DB, Rabello A (2012) Slow clinical improvement after treatment initiation in *Leishmania*/HIV coinfected patients. Rev Soc Bras Med Trop 45: 147-150.

246. Pedrosa Fde A, Ximenes RA (2009) Sociodemographic and environmental risk factors for American cutaneous leishmaniasis (ACL) in the State of Alagoas, Brazil. Am J Trop Med Hyg 81: 195-201.

247. Souza VA, Cortez LR, Dias RA, Amaku M, Ferreira Neto JS, et al. (2012) Space-time cluster analysis of American visceral leishmaniasis in Bauru, Sao Paulo State, Brazil. Cad Saude Publica 28: 1949-1964.

248. Correa Antonialli SA, Torres TG, Paranhos Filho AC, Tolezano JE (2007) Spatial analysis of American Visceral Leishmaniasis in Mato Grosso do Sul State, Central Brazil. J Infect 54: 509-514.

249. Nasser JT, Donalisio MR, Vasconcelos CH (2009) [Spatial distribution of American tegumentary leishmaniasis cases in Campinas, State of Sao Paulo, between 1992 and 2003]. Rev Soc Bras Med Trop 42: 309-314.

250. Werneck GL, Maguire JH (2002) Spatial modeling using mixed models: an ecologic study of visceral leishmaniasis in Teresina, Piaui State, Brazil. Cad Saude Publica 18: 633-637.

251. Tojal da Silva AC, Cupolillo E, Volpini AC, Almeida R, Romero GA (2006) Species diversity causing human cutaneous leishmaniasis in Rio Branco, state of Acre, Brazil. Trop Med Int Health 11: 1388-1398.

252. Mestre GL, Fontes CJ (2007) [The spread of the visceral leishmaniasis epidemic in the State of Mato Grosso, 1998-2005]. Rev Soc Bras Med Trop 40: 42-48.

253. Sampaio RN, Goncalves Mde C, Leite VA, Franca BV, Santos G, et al. (2009) [Study on the transmission of American cutaneous leishmaniasis in the Federal District]. Rev Soc Bras Med Trop 42: 686-690.

254. Gama ME, Costa JM, Gomes CM, Corbett CE (2004) Subclinical form of the American visceral leishmaniasis. Mem Inst Oswaldo Cruz 99: 889-893.

255. Amato VS, Rabello A, Rotondo-Silva A, Kono A, Maldonado TP, et al. (2004) Successful treatment of cutaneous leishmaniasis with lipid formulations of amphotericin B in two immunocompromised patients. Acta Trop 92: 127-132.

256. Almeida RP, Brito J, Machado PL, AR DEJ, Schriefer A, et al. (2005) Successful treatment of refractory cutaneous leishmaniasis with GM-CSF and antimonials. Am J Trop Med Hyg 73: 79-81.

257. Da-Cruz AM, Bittar R, Mattos M, Oliveira-Neto MP, Nogueira R, et al. (2002) T-cell-mediated immune responses in patients with cutaneous or mucosal leishmaniasis: long-term evaluation after therapy. Clin Diagn Lab Immunol 9: 251-256.

258. Posada-Vergara MP, Lindoso JA, Tolezano JE, Pereira-Chioccola VL, Silva MV, et al. (2005) Tegumentary leishmaniasis as a manifestation of immune reconstitution inflammatory syndrome in 2 patients with AIDS. J Infect Dis 192: 1819-1822.

259. Chrusciak-Talhari A, Ribeiro-Rodrigues R, Talhari C, Silva RM, Jr., Ferreira LC, et al. (2009) Tegumentary leishmaniasis as the cause of immune reconstitution inflammatory syndrome in a patient co-infected with human immunodeficiency virus and *Leishmania guyanensis*. Am J Trop Med Hyg 81: 559-564.

260. Frade AF, Oliveira LC, Costa DL, Costa CH, Aquino D, et al. (2011) TGFB1 and IL8 gene polymorphisms and susceptibility to visceral leishmaniasis. Infect Genet Evol 11: 912-916.

261. Rodrigues MZ, Grassi MF, Mehta S, Zhang XQ, Gois LL, et al. (2011) Th1/Th2 cytokine profile in patients coinfected with HIV and *Leishmania* in Brazil. Clin Vaccine Immunol 18: 1765-1769.

262. Cunha JCdL, Lima JWdO, Pompeu MMdL (2006) Transmissão domiciliar de leishmaniose tegumentar e associação entre leishmaniose humana e canina, durante uma epidemia na Serra de Baturité, no estado de Ceará, Brasil. Revista Brasileira de Epidemiologia 9: 425-435.

263. Alexander B, Oliveria EB, Haigh E, Almeida LL (2002) Transmission of *Leishmania* in coffee plantations of Minas Gerais, Brazil. Mem Inst Oswaldo Cruz 97: 627-630.

264. Brustoloni YM, Cunha RV, Consolo LZ, Oliveira AL, Dorval ME, et al. (2010) Treatment of visceral leishmaniasis in children in the Central-West Region of Brazil. Infection 38: 261-267.

265. Franke CR, Staubach C, Ziller M, Schluter H (2002) Trends in the temporal and spatial distribution of visceral and cutaneous leishmaniasis in the state of Bahia, Brazil, from 1985 to 1999. Trans R Soc Trop Med Hyg 96: 236-241.

266. Vera LA, Macêdo VdO, Magalhães AVd, Ciuffo IA, Santos CG, et al. (2002) Úlceras leishmanióticas cutâneas com presença de Corynebacterium diphtheriae. Revista da Sociedade Brasileira de Medicina Tropical 35: 311-313.

267. Ogawa MM, Macedo FS, Alchorne MM, Tomimori-Yamashita J (2002) Unusual location of cutaneous leishmaniasis on the hallux in a Brazilian patient. Int J Dermatol 41: 439-440.

268. Tavora ER, Lasmar EP, Orefice J, Gontijo CM, Andrade Filho JS (2002) Unusual manifestations of leishmaniasis in renal transplant. Transplant Proc 34: 502-503.

269. Lindoso JA, Barbosa RN, Posada-Vergara MP, Duarte MI, Oyafuso LK, et al. (2009) Unusual manifestations of tegumentary leishmaniasis in AIDS patients from the New World. Br J Dermatol 160: 311-318.

270. Bacellar O, Lessa H, Schriefer A, Machado P, Ribeiro de Jesus A, et al. (2002) Up-regulation of Th1-type responses in mucosal leishmaniasis patients. Infect Immun 70: 6734-6740.

271. Silva LMRd, Cunha PR (2007) A urbanização da leishmaniose tegumentar americana no município de Campinas - São Paulo (SP) e região: magnitude do problema e desafios. Anais Brasileiros de Dermatologia 82: 515-519.

272. Albuquerque PL, Silva Junior GB, Freire CC, Oliveira SB, Almeida DM, et al. (2009) Urbanization of visceral leishmaniasis (kala-azar) in Fortaleza, Ceara, Brazil. Rev Panam Salud Publica 26: 330-333.

273. Boaventura VS, de Oliveira JG, Costa JM, Novais FO, de Oliveira CI, et al. (2009) The value of the otorhinolaryngologic exam in correct mucocutaneous leishmaniasis diagnosis. Am J Trop Med Hyg 81: 384-386.

274. Casavechia MTG, Silveira TGV, Teodoro U, Janeiro V, Udo M, et al. Variables associated with the post-treatment healing of lesions in patients with American cutaneous leishmaniasis in Paraná state, Brazil

As variáveis associadas com a cicatrização pós-tratamento de lesões em pacientes com leishmaniose tegumentar americana no estado do Paraná, Brasil. Braz j pharm sci 45: 841-847.

275. Oliveira RA, Silva LS, Carvalho VP, Coutinho AF, Pinheiro FG, et al. (2008) Visceral leishmaniasis after renal transplantation: report of 4 cases in northeastern Brazil. Transpl Infect Dis 10: 364-368.

276. Figueiro-Filho EA, El Beitune P, Queiroz GT, Somensi RS, Morais NO, et al. (2008) Visceral leishmaniasis and pregnancy: analysis of cases reported in a central-western region of Brazil. Arch Gynecol Obstet 278: 13-16.

277. Silva ES, Pacheco RS, Gontijo CM, Carvalho IR, Brazil RP (2002) Visceral leishmaniasis caused by *Leishmania (Viannia) braziliensis* in a patient infected with human immunodeficiency virus. Rev Inst Med Trop Sao Paulo 44: 145-149.

278. Rocha NA, Silva GB, Oliveira MJ, Abreu KL, Franco LF, et al. (2011) Visceral leishmaniasis in children: a cohort of 120 patients in a metropolitan city of Brazil. Turk J Pediatr 53: 154-160.

279. Caldas AJ, Costa JM, Gama ME, Ramos EA, Barral A (2003) Visceral leishmaniasis in pregnancy: a case report. Acta Trop 88: 39-43.

280. Oliveira CM, Oliveira ML, Andrade SC, Girao ES, Ponte CN, et al. (2008) Visceral leishmaniasis in renal transplant recipients: clinical aspects, diagnostic problems, and response to treatment. Transplant Proc 40: 755-760.

281. Marzochi MC, Fagundes A, Andrade MV, Souza MB, Madeira Mde F, et al. (2009) Visceral leishmaniasis in Rio de Janeiro, Brazil: eco-epidemiological aspects and control. Rev Soc Bras Med Trop 42: 570-580.

282. Missawa NA, Borba JF (2009) [Visceral leishmaniasis in the municipality of Varzea Grande, State of Mato Grosso, between 1998 and 2007]. Rev Soc Bras Med Trop 42: 496-502.

283. Queiroz MJ, Alves JG, Correia JB (2004) [Visceral leishmaniasis: clinical and epidemiological features of children in an endemic area]. J Pediatr (Rio J) 80: 141-146.
